# The Effect of Predicted Compliance With a Web-Based Intervention for Anxiety and Depression Among Latin American University Students: Randomized Controlled Trial

**DOI:** 10.2196/64251

**Published:** 2025-02-28

**Authors:** Corina Benjet, Nur Hani Zainal, Yesica Albor, Libia Alvis-Barranco, Nayib Carrasco Tapia, Carlos C Contreras-Ibáñez, Jacqueline Cortés-Morelos, Lorena Cudris-Torres, Francisco R de la Peña, Noé González, Raúl A Gutierrez-Garcia, Eunice Vargas-Contreras, Maria Elena Medina-Mora, Pamela Patiño, Sarah M Gildea, Chris J Kennedy, Alex Luedtke, Nancy A Sampson, Maria V Petukhova, Jose R Zubizarreta, Pim Cuijpers, Alan E Kazdin, Ronald C Kessler

**Affiliations:** 1 Center for Global Mental Health Research National Institute of Psychiatry Ramón de la Fuente Muñiz Mexico City Mexico; 2 Department of Health Care Policy Harvard Medical School Boston, MA United States; 3 Department of Psychology Kent Ridge Campus National University of Singapore Kent Ridge Singapore; 4 Department of Psychology Universidad Popular de Cesar Valledupar Colombia; 5 Department of Psychology Universidad Cooperativa de Colombia Medellin Colombia; 6 Department of Sociology Universidad Autónoma Metropolitana Mexico City Mexico; 7 Department of Psychiatry and Mental Health Universidad Nacional Autónoma de México Mexico City Mexico; 8 Department of Psychology Fundación Universitaria del Area Andina Valledupar Colombia; 9 Unit of Research Promotion National Institute of Psychiatry Ramón de la Fuente Muñiz Mexico City Mexico; 10 Department of Psychology Facultad de Estudios Superiores Universidad La Salle Bajío Salamanca Mexico; 11 Facultad de Ciencias Administrativas y Sociales Universidad Autónoma de Baja California Ensenada Mexico; 12 Seminar of Studies on Globality National Autonomous University of Mexico (UNAM) Mexico City Mexico; 13 Faculty of Psychology National Autonomous University of Mexico (UNAM) Mexico City Mexico; 14 Department of Psychiatry Massachusetts General Hospital Boston, MA United States; 15 Department of Statistics University of Washington Seattle, WA United States; 16 Vaccine and Infectious Disease Division Fred Hutchinson Cancer Research Center Seattle, WA United States; 17 Department of Clinical, Neuro and Developmental Psychology Vrije Universiteit Amsterdam Amsterdam The Netherlands; 18 Department of Psychology Yale University New Haven, CT United States

**Keywords:** anxiety, depression, web-based cognitive behavioral therapy, compliance, randomized controlled trial

## Abstract

**Background:**

Web-based cognitive behavioral therapy (wb-CBT) is a scalable way to reach distressed university students. Guided wb-CBT is typically superior to self-guided wb-CBT over short follow-up periods, but evidence is less clear over longer periods.

**Objective:**

This study aimed to compare short-term (3 months) and longer-term (12 months) aggregate effects of guided and self-guided wb-CBT versus treatment as usual (TAU) in a randomized controlled trial of Colombian and Mexican university students and carry out an initially unplanned secondary analysis of the role of differential predicted compliance in explaining these differences.

**Methods:**

The 1319 participants, recruited either through email and social media outreach invitations or from waiting lists of campus mental health clinics, were undergraduates (1038/1319, 78.7% female) with clinically significant baseline anxiety (Generalized Anxiety Disorder–7 score≥10) or depression (Patient Health Questionnaire–9 score≥10). The intervention arms comprised guided wb-CBT with weekly asynchronous written human feedback, self-guided wb-CBT with the same content as the guided modality, and TAU as provided at each university. The prespecified primary outcome was joint remission (Generalized Anxiety Disorder–7 score=0-4 and Patient Health Questionnaire–9 score=0-4). The secondary outcome was joint symptom reduction (mean scores on the Patient Health Questionnaire Anxiety and Depression Scale) at 3 and 12 months after randomization.

**Results:**

As reported previously, 3-month outcomes were significantly better with guided wb-CBT than self-guided wb-CBT (*P*=.02) or TAU (*P*=.02). However, subsequent follow-up showed that 12-month joint remission (adjusted risk differences=6.0-6.5, SE 0.4-0.5, and *P*<.001 to *P*=.007; adjusted mean differences=2.70-2.69, SE 0.7-0.8, and *P*<.001 to *P*=.001) was significantly better with self-guided wb-CBT than with the other interventions. Participants randomly assigned to the guided wb-CBT arm spent twice as many minutes logged on as those in the self-guided wb-CBT arm in the first 12 weeks (mean 12.5, SD 36.9 vs 5.9, SD 27.7; *χ*^2^_1_=107.1, *P*<.001), whereas participants in the self-guided wb-CBT arm spent twice as many minutes logged on as those in the guided wb-CBT arm in weeks 13 to 52 (mean 0.4, SD 7.5 vs 0.2, SD 4.4; *χ*^2^_1_=10.5, *P*=.001). Subgroup analysis showed that this longer-term superiority of self-guided wb-CBT was confined to the 40% (528/1319) of participants with high predicted self-guided wb-CBT compliance beyond 3 months based on a counterfactual nested cross-validated machine learning model. The 12-month outcome differences were nonsignificant across arms among other participants (all *P*>.05).

**Conclusions:**

The results have important practical implications for precision intervention targeting to maximize longer-term wb-CBT benefits. Future research needs to investigate strategies to increase sustained guided wb-CBT use once guidance ends.

**Trial Registration:**

ClinicalTrials.gov NCT04780542; https://www.clinicaltrials.gov/study/NCT04780542

**International Registered Report Identifier (IRRID):**

RR2-10.1186/s13063-022-06255-3

## Introduction

### Background

Web-based cognitive behavioral therapy (wb-CBT) has been suggested as a promising strategy for increasing the scalability, reach, and affordability of mental health services for clinically significant anxiety and depression [1-3], especially in populations with internet access and literacy, such as university students [4,5], and for populations with limited availability of in-person mental health services, such as those in Latin America [6]. wb-CBT has generally been found to be as effective as face-to-face cognitive behavioral therapy (CBT) [7-10]. However, uptake and engagement have been important challenges [11].

wb-CBTs vary in whether they are *guided*, in which case human support is provided by a professional or trained lay supporter (eg, phone calls or messages) to encourage compliance, or *self-guided*, in which case no human support is provided. Guided modalities are generally found to be more effective than self-guided modalities [12], although a recent meta-analysis suggests that this might be less true in low- and middle-income countries (LMICs) due to self-guided wb-CBT having effect sizes as high as those of guided wb-CBT [13]. Treatment compliance is generally higher in guided than in self-guided modalities and is considered the main reason why guided wb-CBT usually yields better outcomes than self-guided wb-CBT [14]. Broad interest exists in improving uptake and effectiveness of self-guided wb-CBT given its much lower cost and great potential for scalability, reach, and affordability. In addition, there is interest in determining whether some subset of participants might be helped as much by self-guided as by guided wb-CBT or more and, if so, developing a precision treatment rule that targets assignment of the different modalities in a way that maximizes clinical benefit at the lowest possible cost [15].

We implemented a 3-armed wb-CBT trial with Colombian and Mexican university students who met criteria at baseline for clinically significant anxiety or depression to investigate the possibility of developing a precision treatment rule for optimal assignment of guided and self-guided wb-CBT. In previous reports, we showed that, consistent with earlier research, aggregate effects in increasing joint anxiety-depression remission and reducing symptoms were significantly better for guided wb-CBT than for either self-guided wb-CBT or treatment as usual (TAU) after 3 months [16] but that there was significant heterogeneity in these average effects such that close to one-third of participants benefited as much from self-guided as from guided wb-CBT or more [17] over this same follow-up period. This is a potentially important result because it provides a principled basis for optimizing the joint use of guided and self-guided wb-CBT.

However, a limitation of our work so far is that only short-term effects (ie, 3 months after randomization) have been examined. This limitation is shared by the larger wb-CBT literature, which tells us much less about longer-term than short-term effects. In a recent meta-analysis of all randomized controlled trials evaluating the comparative effects of guided and self-guided wb-CBT on common mental disorders over ≥12 months, we found no consistent difference in the longer-term effects of guided compared to self-guided wb-CBT, although both modalities continued to be associated with significantly better outcomes than controls [18]. However, importantly, none of the studies in this meta-analysis came from LMICs.

### Objectives

This report presents the first results of a controlled trial in LMICs to compare the effects of guided and self-guided wb-CBT 12 months after randomization. We focused on a sample of Colombian and Mexican university students with anxiety or depression. We also expanded our earlier preplanned investigation of heterogeneity in comparative intervention effects with a secondary analysis focused on determining whether a subset of participants can be identified at baseline (ie, before randomization) who would have equal or better longer-term outcomes with self-guided compared to guided wb-CBT and the extent to which differential long-term intervention compliance might account for such heterogeneity.

## Methods

### Sample and Procedures

Participants were 1319 undergraduate students (n=1038, 78.7% female; median age 21, IQR 19-22 years) from 7 universities in Colombia and Mexico who were recruited either through email and social media outreach invitations or from waiting lists of campus mental health clinics in the universities that had such clinics. Inclusion criteria were completing the baseline assessment and reporting clinically significant anxiety (Generalized Anxiety Disorder–7 [GAD-7] scores of ≥10) [19] or depression (Patient Health Questionnaire–9 [PHQ-9] scores of ≥10) [20] and consenting to be randomly assigned to guided wb-CBT, self-guided wb-CBT, or TAU. Exclusion criteria were reporting recent suicidal ideation with intent or screening positive for a history of bipolar disorder or nonaffective psychosis. Students who were excluded from the trial were contacted by a clinical liaison at their university and provided with appropriate referrals.

Study enrollment took place between March 1, 2021, and October 26, 2021. Eligible students were block randomly assigned with equal allocation and stratification by sex and severity of baseline anxiety and depression across the 3 intervention arms. In total, 2 follow-up web-based self-administered questionnaires (SAQs) were administered 3 and 12 months after randomization to assess changes in symptom scores. Initial follow-up SAQ nonrespondents were sent email reminders and Telegram (Telegram FZ-LLC) or WhatsApp (Meta Platforms) messages and phoned to reduce loss to follow-up. Baseline participants who did not respond to the 3-month follow-up SAQ were still sent the 12-month follow-up SAQ and reminders. Further information about trial design has been published elsewhere [16]. The trial was preregistered at ClinicalTrials.gov (NCT04780542).

### Interventions

The wb-CBT program (both guided and self-guided modalities) was a culturally adapted version of SilverCloud Health by Amwell’s Space from Depression and Anxiety program, a transdiagnostic wb-CBT program that can be accessed via computer, tablet, or mobile phone and that has been found to be effective in treating anxiety and depression [21-23]. The content is identical in the guided and self-guided programs, but participants assigned to the guided program receive weekly asynchronous written messages during the first 8 weeks after randomization through the platform from trained Bachelor of Arts–level coaches with a degree in psychology intended to generate personalized experiences and offer feedback [24]. The intervention has 7 primary modules that focus on cognitive restructuring, behavioral activation, and relaxation techniques and several other ancillary modules (eg, sleep and anger). The program content, which includes videos, audios, exercises, and vignettes, is intended for completion within 8 weeks, although participants have continued access to the program for initial use or refresher reviews for 12 months after randomization (described in more detail in the study by Benjet et al [16]). TAU consisted of referral to the clinic in the 3 universities that had student mental health clinics and referral to informal counseling services that faculty provide in the other universities to place students with anxiety or depression with community treatment providers. As randomization occurred during the COVID-19 pandemic lockdown, most university services were offered solely through videoconferencing platforms during the study. We chose to compare to TAU rather than to an active or waitlist control condition to determine the extent to which these interventions improved upon the services actually available in the participating universities.

### Measures

#### Anxiety and Depression

Anxiety was measured using the self-report GAD-7 [19], and depression was measured using the self-report PHQ-9 [20], both of which are commonly used in psychotherapy trials, have good psychometric properties [25], and have been shown previously to have strong convergent validity with other anxiety and depression scales in Colombia [26,27] and Mexico [28,29]. These scales are often combined into a single scale to measure anxiety and depression in the Patient Health Questionnaire Anxiety and Depression Scale (PHQ-ADS [25]). We examined joint remission (the conjunction of GAD-7 score=0-4 and PHQ-9 score=0-4) as well as mean PHQ-ADS scores at 3 and 12 months.

#### Intervention Compliance

Metadata from the web-based SilverCloud Health intervention portal provided use metrics of compliance. Such compliance metrics are often used in analyses of web-based intervention trials [30,31]. We focused on the number of minutes that each participant spent logged on each week after the end of the guidance phase of the intervention over the course of 12 months as our primary measure of compliance as adequate time has been a consistent predictor of higher adherence to wb-CBTs and other web-based mental health interventions [32].

#### Baseline Covariates

The baseline survey contained a wide range of potential predictors of heterogeneity in intervention effects. These predictors fall into 11 domains: sociodemographics, university-related factors, stressors related to COVID-19, other recent and lifetime stressors, anxiety and depression characteristics, comorbid mental disorders, mental health treatment, physical health, social networks and supports, personality or temperament and psychological resilience, and internet literacy and preferences (these predictors are detailed in the study by Benjet et al [16]).

### Data Analysis

#### Average Treatment Effects

All average treatment effect (ATE) analyses incorporated adjustments for loss to follow-up using a doubly robust estimation method that combined outcome modeling (similar to imputing missing values) and propensity score modeling through the longitudinal targeted minimum loss-based estimation method [33]. For the 3-month analysis, we used the *tmle3* R package (R Foundation for Statistical Computing), whereas for the 12-month analysis, we used *ltmle* [34,35]. The latter program allows information about partial follow-up (in our case, response to the 3-month follow-up but not to the 12-month follow-up) to be used in adjusting for loss to follow-up. We used the *mice* R package [36,37] for multiple imputation with predictive mean matching to predict 3-month follow-up results for participants without 3-month data but with 12-month data.

Our primary outcome, joint anxiety-depression remission, was defined as follow-up scores of 0 to 4 on both the GAD-7 and PHQ-9. The analysis estimated these joint remission rates by adjusting for nonrandom loss to follow-up and then calculated adjusted risk differences (ARDs) across arms. In our analysis of mean symptom changes, in comparison, we estimated baseline and 3- and 12-month means, again adjusting for loss to follow-up, and then calculated adjusted mean differences (AMDs) across arms.

#### Heterogeneity in Intervention Effects Due to Differential Compliance

We noted in the *Introduction* section that the stronger effects typically found for guided than for self-guided wb-CBT are usually interpreted as being due to the higher compliance of participants randomly assigned to guided wb-CBT than to self-guided wb-CBT. However, there is often no test of this assumption in wb-CBT trials, and when such tests are carried out, they are typically based on conventional per-protocol or as-treated analysis methods, both of which are biased because of their failure to adjust for nonrandom determinants of compliance [38]. More sophisticated instrumental variable (IV) analysis methods with randomization treated as the instrument are sometimes used to address the fact that compliance can only be observed after randomization. However, IV analysis assumes that a principled dichotomous measure of compliance exists that captures the full effect of randomization (eg, that the patient randomly assigned to receive a vaccination is either vaccinated or not vaccinated) [39]. This assumption is not met in psychotherapy trials, where compliance is ordinal rather than dichotomous (ie, participants differ in the number of times they log on and the number of minutes they spend with the intervention each time they log on, among other things). Selecting an arbitrary dichotomization of such ordinal measures for purposes of IV analysis will typically yield biased estimates of the effects of compliance.

It is possible to overcome these problems by estimating more complex IV models that are designed specifically to model dose-response effects [40]. However, this approach makes use of sophisticated doubly robust estimation methods that attempt to adjust for uncontrolled predictors of compliance that also have independent influences on the outcome [41]. Valid use of these methods requires that postrandomization measures are available for the informative predictors of compliance, which is typically not the case. We used a different approach as we did not have postrandomization measures to predict compliance. In this approach, given our interest in heterogeneity, we used the rich set of baseline covariates in our trial to develop a machine learning model that predicted observed compliance among participants randomly assigned to self-guided wb-CBT. We did this using the *SuperLearner* R program [42] to train a nested 10-fold–cross-validated ensemble machine learning model. Given that the baseline assessment was obtained for all participants across all 3 arms, individual-level predicted compliance scores could then be generated for all participants in the trial regardless of the arm to which they were randomly assigned. This allowed us to define predicted compliance based on information available before randomization and make sure that individual-level scores in the self-guided arm were not biased by knowledge of the participants’ observed compliance by virtue of the use of nested 10-fold cross-validation. Predicted compliance was then used as a specifier in evaluating comparative intervention effects in subgroups defined by the likelihood that participants would continue to comply with self-guided wb-CBT over the full 12-month follow-up period if they were assigned to it. This approach has been recommended as the best way to study baseline predictors of heterogeneity based on differential compliance in cases in which heterogeneity is found [43].

The kernel Shapley additive explanations (SHAP) method [44], applied through the *fastshap* R package [45], was then used to examine which baseline predictors played the largest role in defining predicted compliance. SHAP values are created by calculating, one at a time for each significant predictor, the extent to which predicted outcome scores change under the prediction model when the score on the predictor is changed from its observed value to the mean value in the sample. The SHAP value for a given predictor is defined as the mean of the absolute value of this difference across all participants. We report the proportional SHAP value (SHAP_P_), the SHAP value for the individual predictor divided by the SHAP value for the entire model (ie, the effect of setting all predictors to their mean values for all participants). SHAP_P_ values for individual predictors can add up to >100% because most people have values for some predictors above the mean and others have values below the mean.

### Ethical Considerations

This study protocol was reviewed and approved by the institutional review boards of the Instituto Nacional de Psiquiatría (National Institute of Psychiatry) Ramón de la Fuente Muñiz in Mexico (approval CEI/C/015/2020) and Harvard Medical School in the United States (approval 20-1494). Web-based informed consent was obtained from participants. The data were deidentified, and participants received a gift card equivalent to approximately US $20 for each follow-up SAQ.

## Results

### Participant Enrollment, Allocation, and Completion

**Figure 1:**
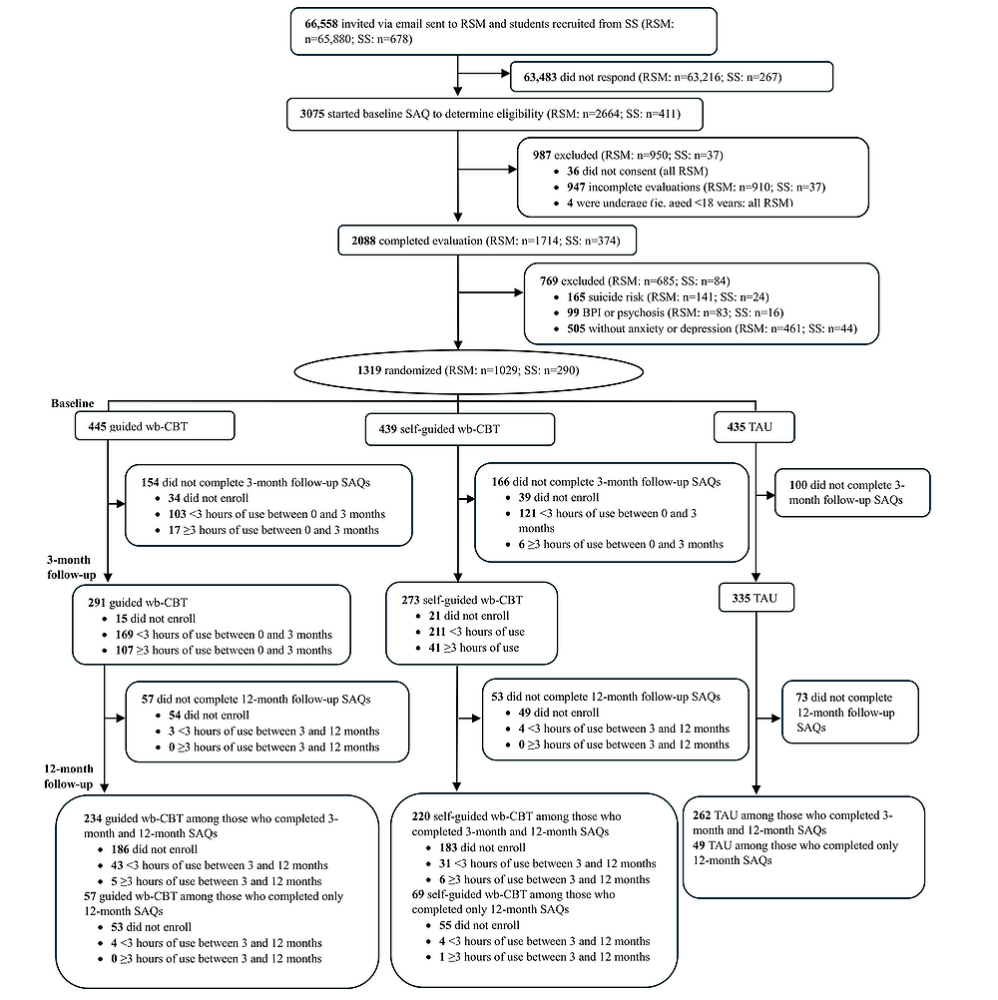
CONSORT (Consolidated Standards of Reporting Trials) flow diagram. “Did not enroll” refers to participants who completed the baseline self-administered questionnaire (SAQ) but did not ever log in to start either the guided or self-guided web-based cognitive behavioral therapy (wb-CBT). “Guided wb-CBT among those who completed only 3-month and 12-month SAQs” and “Guided wb-CBT among those who completed only 12-month SAQs” refer to participants who were initially randomly assigned to guided wb-CBT at baseline and no longer had a guide to encourage them to engage with wb-CBT from the 3- to 12-month follow-ups. BPI: Bipolar I; RSM: representative samples or social media; SS: student services; TAU: treatment as usual.

Figure 1 shows the CONSORT (Consolidated Standards of Reporting Trials) diagram of the enrollment, allocation, and 3- and 12-month follow-up rates of trial participants. The completed CONSORT checklist can be found in Multimedia Appendix 1.

Table 1 presents the patterns and selected baseline predictors of completing the 3- and 12-month follow-up SAQs among the 1319 participants who completed the baseline SAQ and were randomly assigned. A total of 54.3% (716/1319) completed both the 3- and 12-month postrandomization SAQs, and 80.7% (1065/1319) completed at least one follow-up SAQ (183/1319, 13.9% completed only the 3-month SAQ, and 166/1319, 12.6% completed only the 12-month SAQ), whereas the remaining 19.3% (254/1319) failed to complete either.

**Table 1:** Patterns and selected baseline predictors of completing follow-up self-administered questionnaires (SAQs) among the participants who completed the baseline SAQ (N=1319)^a^.

		Completed both 3- and 12-month SAQs, n (%)		Completed 3-month SAQ (regardless of 12-month completion status), n (%)		Completed 12-month SAQ (regardless of 3-month completion status), n (%)
Participants		716 (54.3)		899 (68.2)		882 (66.9)
**Intervention arm^b^**						
	Guided (n=445)	234 (52.6)	291 (65.4)		291 (65.4)	
	Self-guided (n=439)	220 (50.1)	273 (62.2)		280 (63.8)	
	TAU^c^ (n=435)	262 (60.2)	335 (77)		311 (71.5)	
**Country^d,e^**						
	Colombia (n=594)	285 (48)	338 (56.9)		403 (67.8)	
	Mexico (n=725)	431 (59.4)	561 (77.4)		479 (66.1)	
**Sex^f,g^**						
	Male (n=281)	147 (52.3)	184 (65.5)		179 (63.7)	
	Female (n=1038)	569 (54.8)	715 (68.9)		703 (67.7)	
**Sexual orientation^h,i^**						
	Heterosexual (n=916)	481 (52.5)	610 (66.6)		604 (65.9)	
	Gay or lesbian (n=61)	32 (52.5)	39 (63.9)		41 (67.2)	
	Bisexual (n=196)	111 (56.6)	141 (71.9)		135 (68.9)	
	Other (asexual, unsure, or “other”; n=146)	92 (63)	109 (74.7)		102 (69.9)	
**Age group (y)^j,k^**						
	18-19 (n=354)	196 (55.4)	247 (69.8)		238 (67.2)	
	20 (n=251)	138 (55)	177 (70.5)		161 (64.1)	
	21-22 (n=397)	222 (55.9)	278 (70)		269 (67.8)	
	≥23 (n=317)	160 (50.5)	197 (62.1)		214 (67.5)	
**First-generation university student^l,m^**						
	Yes (n=738)	394 (53.4)	485 (65.7)		486 (65.9)	
	No (n=581)	322 (55.4)	414 (71.3)		396 (68.2)	
**Did the university have a mental health clinic?^n,o^**						
	Yes, and the students were recruited from the waiting list (n=290)	183 (63.1)	237 (81.7)		197 (67.9)	
	Yes, but the students were recruited from the student body (n=607)	331 (54.5)	415 (68.4)		417 (68.7)	
	No (all students were recruited from the student body; n=422)	202 (47.9)	247 (58.5)		268 (63.5)	
**Severity of anxiety (GAD-7^p^)^q,r^**						
	Severe (n=494)	258 (52.2)	335 (67.8)		318 (64.4)	
	Moderate (n=434)	247 (56.9)	300 (69.1)		301 (69.4)	
	Mild or none (n=391)	211 (54)	264 (67.5)		263 (67.3)	
**Severity of depression (PHQ-9^s^)^t,u^**						
	Severe (n=488)	259 (53.1)	335 (68.6)		329 (67.4)	
	Moderate (including moderate-severe; n=702)	381 (54.3)	466 (66.4)		462 (65.8)	
	Mild or none (n=129)	76 (58.9)	98 (76)		91 (70.5)	
**Comorbidity^v,w^**						
	Severe^x^ on both the GAD-7 and PHQ-9 (n=282)	144 (51.1)	190 (67.4)		187 (66.3)	
	Severe^x^ on one and moderate on the other (n=284)	153 (53.9)	189 (66.5)		184 (64.8)	
	Severe^x^ on one and mild to none on the other (n=134)	76 (56.7)	101 (75.4)		89 (66.4)	
	Moderate on both (n=233)	132 (56.7)	158 (67.8)		157 (67.4)	
	Moderate on one and mild to none on the other (n=386)	211 (54.7)	261 (67.6)		265 (68.7)	

^a^*P* values correspond to tests of the significance of the associations between baseline predictors and completing the follow-up SAQ in the subgroup.

^b^Chi-square test across intervention categories within the subgroup—completed both 3- and 12-month SAQs: *χ*^2^_2_=9.7, *P*=.008; completed 3-month SAQ: *χ*^2^_2_=24.0, *P*<.001; completed 12-month SAQ: *χ*^2^_2_=6.5, *P*=.04.

^c^TAU: treatment as usual.

^d^Main effect chi-square test—completed both 3- and 12-month SAQs: *χ*^2^_1_=17.5, *P*<.001; completed 3-month SAQ: *χ*^2^_1_=63.9, *P*<.001; completed 12-month SAQ: *χ*^2^_1_=0.5, *P*=.50.

^e^Heterogeneity chi-square test—completed both 3- and 12-month SAQs: *χ*^2^_2_=0.7, *P*=.69; completed 3-month SAQ: *χ*^2^_2_=0.6, *P*=.75; completed 12-month SAQ: *χ*^2^_2_=1.4, *P*=.49.

^f^Main effect chi-square test—completed both 3- and 12-month SAQs: *χ*^2^_1_=0.6, *P*=.46; completed 3-month SAQ: *χ*^2^_1_=1.1, *P*=.29; completed 12-month SAQ: *χ*^2^_1_=1.6, *P*=.21.

^g^Heterogeneity chi-square test—completed both 3- and 12-month SAQs: *χ*^2^_2_=0.3, *P*=.86; completed 3-month SAQ: *χ*^2^_2_=0.2, *P*=.90; completed 12-month SAQ: *χ*^2^_2_=0.8, *P*=.66.

^h^Main effect chi-square test—completed both 3- and 12-month SAQs: *χ*^2^_3_=6.3, *P*=.10; completed 3-month SAQ: *χ*^2^_3_=0.6, *P*=.11; completed 12-month SAQ: *χ*^2^_3_=1.3, *P*=.72.

^i^Heterogeneity chi-square test—completed both 3- and 12-month SAQs: *χ*^2^_6_=10.4, *P*=.11; completed 3-month SAQ: *χ*^2^_6_=16.3, *P*=.01; completed 12-month SAQ: *χ*^2^_6_=5.5, *P*=.48 (refer to Multimedia Appendix 2 for more details on the significant interaction between intervention arms and sexual orientation to predict 3-month SAQ completion status [regardless of 12-month SAQ completion status]).

^j^Main effect chi-square test—completed both 3- and 12-month SAQs: *χ*^2^_3_=2.5, *P*=.48; completed 3-month SAQ: *χ*^2^_3_=6.6, *P*=.09; completed 12-month SAQ: *χ*^2^_3_=1.0, *P*=.79.

^k^Heterogeneity chi-square test—completed both 3- and 12-month SAQs: *χ*^2^_6_=4.8, *P*=.58; completed 3-month SAQ: *χ*^2^_6_=4.2, *P*=.66; completed 12-month SAQ: *χ*^2^_6_=8.0, *P*=.24.

^l^Main effect chi-square test—completed both 3- and 12-month SAQs: *χ*^2^_1_=0.5, *P*=.46; completed 3-month SAQ: *χ*^2^_1_=4.7, *P*=.03; completed 12-month SAQ: *χ*^2^_1_=0.8, *P*=.38.

^m^Heterogeneity chi-square test—completed both 3- and 12-month SAQs: *χ*^2^_2_=0.3, *P*=.86; completed 3-month SAQ: *χ*^2^_2_=1.7, *P*=.42; completed 12-month SAQ: *χ*^2^_2_=0.9, *P*=.63.

^n^Main effect chi-square test—completed both 3- and 12-month SAQs: *χ*^2^_2_=16.4, *P*<.001; completed 3-month SAQ: *χ*^2^_2_=48.0, *P*<.001; completed 12-month SAQ: *χ*^2^_2_=3.1, *P*=.21.

^o^Heterogeneity chi-square test—completed both 3- and 12-month SAQs: *χ*^2^_4_=0.4, *P*=.98; completed 3-month SAQ: *χ*^2^_4_=4.9, *P*=.30; completed 12-month SAQ: *χ*^2^_4_=2.0, *P*=.74.

^p^GAD-7: Generalized Anxiety Disorder–7. *Severe* refers to GAD-7≥15, *moderate* refers to GAD-7=10-14, and *mild or none* refers to GAD-7=0-9.

^q^Main effect chi-square test—completed both 3- and 12-month SAQs: *χ*^2^_2_=2.1, *P*=.36; completed 3-month SAQ: *χ*^2^_2_=0.3, *P*=.87; completed 12-month SAQ: *χ*^2^_2_=2.6, *P*=.27.

^r^Heterogeneity chi-square test—completed both 3- and 12-month SAQs: *χ*^2^_4_=0.4, *P*=.99; completed 3-month SAQ: *χ*^2^_4_=0.4, *P*=.98; completed 12-month SAQ: *χ*^2^_4_=1.4, *P*=.84.

^s^PHQ-9: Patient Health Questionnaire–9. Severe refers to PHQ-9≥20, moderately severe refers to PHQ-9=15-19, moderate refers to PHQ-9=10-14, and mild or none refers to PHQ-9=0-9.

^t^Main effect chi-square test—completed both 3- and 12-month SAQs: *χ*^2^_2_=1.4, *P*=.49; completed 3-month SAQ: *χ*^2^_2_=5.2, *P*=.07; completed 12-month SAQ: *χ*^2^_2_=1.2, *P*=.54.

^u^Heterogeneity chi-square test—completed both 3- and 12-month SAQs: *χ*^2^_4_=1.4, *P*=.84; completed 3-month SAQ: *χ*^2^_4_=3.9, *P*=.43; completed 12-month SAQ: *χ*^2^_4_=1.6, *P*=.80.

^v^Main effect chi-square test—completed both 3- and 12-month SAQs: *χ*^2^_4_=2.1, *P*=.72; completed 3-month SAQ: *χ*^2^_4_=4.1, *P*=.39; completed 12-month SAQ: *χ*^2^_4_=1.2, *P*=.88.

^w^Heterogeneity chi-square test—completed both 3- and 12-month SAQs: *χ*^2^_8_=3.5, *P*=.90; completed 3-month SAQ: *χ*^2^_8_=3.4, *P*=.90; completed 12-month SAQ: *χ*^2^_8_=4.3, *P*=.83.

^x^Including either severe or moderately severe scores on the PHQ-9.

### Sample Distribution

Distributions of clinical, demographic, and university characteristic variables were comparable across arms (Table S1 in Multimedia Appendix 2).

### Patterns and Predictors of SAQ Completion

A significantly higher joint 3- and 12-month follow-up SAQ completion rate was observed in TAU (262/435, 60.2%) than in guided (234/445, 52.6%) or self-guided (220/439, 50.1%) wb-CBT (*χ*^2^_2_=9.7, *P*=.008) in Mexico (431/725, 59.4%) than in Colombia (285/594, 48%; *χ*^2^_2_=17.5, *P*<.001) and in the clinic waitlist subsample (183/290, 63.1%) than in the general student body subsample (553/1029, 53.7%; *χ*^2^_2_=48.0, *P*<.001; Table 1). The associations of baseline variables with joint follow-up SAQ completion did not differ significantly (*P*=.11-.99) across intervention arms in this subgroup.

Parallel analyses were carried out of 3-month SAQ completion regardless of 12-month completion (899/1319, 68.2%) and of 12-month SAQ completion regardless of 3-month completion (882/1319, 66.9%). Significant differences in completion rates by baseline variables such as those for joint completion were found in both analyses, with general comparability of associations between baseline variables and completion across intervention arms (Table 1). The single exception was a significant interaction between sexual orientation and intervention arm in predicting 3-month SAQ completion (*χ*^2^_6_=16.3, *P*=.01; Table S2 in Multimedia Appendix 2). Heterosexual, gay or lesbian, and bisexual individuals were more likely to complete the SAQs if assigned to TAU (13/18, 72%-55/61, 90%) than self-guided (43/80, 54%-11/17, 65%) and guided (15/26, 58%-43/55, 78%) wb-CBT, whereas participants who identified with *other* sexual orientations were more likely to complete the SAQs if assigned to self-guided wb-CBT (39/49, 80%) than the other 2 arms (40/56, 71%-30/41, 73%). Importantly, the association of predicted self-guided wb-CBT compliance with both 3- and 12-month SAQ completion was comparable across intervention arms (Table S3 in Multimedia Appendix 2), and the association of 3-month remission with 12-month SAQ completion in the subsample of 3-month SAQ completers was comparable across intervention arms (Table S3 in Multimedia Appendix 2).

### ATEs at 3 and 12 Months

Joint anxiety-depression remission rates differed significantly across intervention arms at both 3 months (37.2%-50.3%; *χ*^2^_2_=12.4, *P*=.002) and 12 months (32.5%-38.9%; *χ*^2^_2_=7.6, *P*=.02; Table 2). As we reported in a previous publication [16], at 3 months, the remission rate of guided wb-CBT was significantly higher than that of either self-guided wb-CBT (ARD=13.1%; *χ*^2^_1_=10.4, *P*=.001) or TAU (ARD=11.2%; *χ*^2^_1_=8.4, *P*=.004), but the remission rates were nonsignificantly different between self-guided wb-CBT and TAU (ARD=−1.9%; *χ*^2^_2_=0.2, *P*=.63). The new results for 12 months reported in this paper for the first time, in comparison, show that the remission rate of self-guided wb-CBT was significantly higher than that of either guided wb-CBT (ARD=−6%; *χ*^2^_1_=4.9, *P*=.03) or TAU (ARD=−6.5%; *χ*^2^_1_=6.3, *P*=.01), whereas the remission rates were nonsignificantly different between guided wb-CBT and TAU (ARD=0.4%; *χ*^2^_1_=0.0, *P*=.86).

**Table 2:** Average treatment effects across arms at 3 and 12 months and adjusted risk differences (ARDs) for the joint remission outcome (N=1319).

			Total					Guided (n=445)					Self-guided (n=439)					TAU^a^ (n=435)	
			Estimate^b^ (SE)		*P* value		Estimate (SE)			*P* value		Estimate (SE)			*P* value		Estimate (SE)		*P* value
**Joint remission on the GAD-7^c^ and PHQ-9^d,e^**																			
	3 months^f^	42.2 (5.0)		<.001		50.3 (2.9)			<.001		37.2 (2.9)			<.001		39.0 (2.6)			<.001
	12 months^g^	35.0 (3.0)		<.001		33.0 (1.9)			<.001		38.9 (1.9)			<.001		32.5 (1.7)			<.001

^a^TAU: treatment as usual.

^b^Estimate of joint remission rates on both the Generalized Anxiety Disorder–7 and Patient Health Questionnaire–9 or means of Patient Health Questionnaire Anxiety and Depression Scale scores.

^c^GAD-7: Generalized Anxiety Disorder–7 (0-21).

^d^PHQ-9: Patient Health Questionnaire–9 (0-29).

^e^Joint remission was defined as scores of 0 to 4 on both the GAD-7 and PHQ-9.

^f^Guided versus self-guided web-based cognitive behavioral therapy (wb-CBT): ARD=13.1 (SE 4.1) and *P*=.001; TAU versus self-guided wb-CBT: ARD=1.9 (SE 3.9) and *P*=.63; guided wb-CBT versus TAU: ARD=11.2 (SE 3.9) and *P*=.004; self-guided wb-CBT versus TAU: ARD=–1.9 (SE 3.9) and *P*=.63. The 2-sided test with 2 *df* at the .05 level for the overall variations across the 3 arms on joint remission on the GAD-7 and PHQ-9 was statistically significant at 3 months (*χ*^2^_2_=12.4, *P*=.002) and 12 months (*χ*^2^_2_=7.6, *P*=.02).

^g^Guided versus self-guided wb-CBT: ARD=–6.0 (SE 2.7) and *P*=.03; TAU versus self-guided wb-CBT: ARD=–6.5 (SE 2.6) and *P*=.01; guided wb-CBT versus TAU: ARD=0.4 (SE 2.6) and *P*=.88; self-guided wb-CBT versus TAU: ARD=6.5 (SE 2.6) and *P*=.01. The 2-sided test with 2 *df* at the .05 level for the overall variations across the 3 arms on joint remission on the GAD-7 and PHQ-9 was statistically significant at 3 months (*χ*^2^_2_=12.4, *P*=.002) and 12 months (*χ*^2^_2_=7.6, *P*=.02).

Comparative intervention effects were broadly similar for mean PHQ-ADS scores. These scores were significantly lower at both the 3- (13.1-15.6) and 12-month (15.1-17.8) follow-ups than at baseline (28.4-28.7) in all arms but varied significantly across arms at both 3 months (*χ*^2^_2_=8.0, *P*=.02) and 12 months (*χ*^2^_2_=19.5, *P*<.001; Table 3). At 3 months, the mean PHQ-ADS score was significantly lower for guided wb-CBT than for both self-guided wb-CBT (AMD=−1.9; *χ*^2^_1_=4.2, *P*=.04) and TAU (AMD=−2.4; *χ*^2^_1_=7.3, *P*=.007), whereas mean PHQ-ADS scores were nonsignificantly different between self-guided wb-CBT and TAU (AMD=−0.5; *χ*^2^_1_=0.4, *P*=.55). At 12 months, in comparison, the mean PHQ-ADS scores were significantly lower for self-guided wb-CBT than for both guided wb-CBT (AMD=2.7; *χ*^2^_1_=11.5, *P*=.001) and TAU (AMD=−2.7; *χ*^2^_1_=15.3, *P*<.001), whereas mean PHQ-ADS scores were nonsignificantly different between guided wb-CBT and TAU (AMD=0.0; *χ*^2^_1_=0.0, *P*=.99).

**Table 3:** Average treatment effects across arms at 3 and 12 months and adjusted mean differences (AMDs) for the Patient Health Questionnaire Anxiety and Depression Scale (PHQ-ADS) outcome (N=1319).

		Total			Guided (n=445)			Self-guided (n=439)			TAU^a^ (n=435)	
		Estimate (SE)	*P* value	Estimate (SE)		*P* value	Estimate (SE)		*P* value	Estimate (SE)		*P* value

**Mean PHQ-ADS scores**												
	Baseline	28.6 (1.0)	<.001	28.7 (1.0)		<.001	28.4 (1.0)		<.001	28.7 (0.9)		<.001
	3 months^b^	14.6 (0.7)	<.001	13.1 (0.7)		<.001	15.0 (0.7)		<.001	15.6 (0.6)		<.001
	12 months^c^	16.9 (0.9)	<.001	17.8 (0.6)		<.001	15.1 (0.5)		<.001	17.8 (0.5)		<.001

^a^TAU: treatment as usual.

^b^Guided versus self-guided web-based cognitive behavioral therapy (wb-CBT): AMD=–1.9 (SE 0.9) and *P*=.04; TAU versus self-guided wb-CBT: AMD=0.5 (SE 0.9) and *P*=.58; guided wb-CBT versus TAU: AMD=–2.4 (SE 0.9) and *P*=.008; self-guided wb-CBT versus TAU: AMD=–0.5 (SE 0.9) and *P*=.58. The 2-sided test with 2 *df* at the .05 level for the overall variations across the 3 arms on mean PHQ-ADS scores was statistically significant at 3 months (*χ*^2^_2_=8.0, *P*=.02) and 12 months (*χ*^2^_2_=19.5, *P*<.001) but not at baseline (*χ*^2^_2_=0.0, *P*>.99).

^c^Guided versus self-guided wb-CBT: AMD=2.7 (SE 0.8) and *P*=.001; TAU versus self-guided wb-CBT: AMD=2.7 (SE 0.7) and *P*<.001; guided wb-CBT versus TAU: AMD=0.0 (SE 0.8) and *P*>.99; self-guided wb-CBT versus TAU: AMD=–2.7 (SE 0.7) and *P*<.001. The 2-sided test with 2 *df* at the .05 level for the overall variations across the 3 arms on mean PHQ-ADS scores was statistically significant at 3 months (*χ*^2^_2_=8.0, *P*=.02) and 12 months (*χ*^2^_2_=19.5, *P*<.001) but not at baseline (*χ*^2^_2_=0.0, *P*>.99).

### Observed Compliance at 3 and 12 Months

Could the ATE findings be attributed to observed differences in compliance? As noted previously in the *Methods* section, we initially addressed this question by focusing on the metadata for time spent with the intervention. From weeks 1 to 12, both average minutes per week (12.5, SD 36.9 for guided vs 5.9, SD 27.7 for self-guided wb-CBT; *χ*^2^_1_=107.1, *P*<.001) and the proportions of participants who spent ≥5, ≥10, and ≥30 minutes per week with the intervention (*χ*^2^_1_=171.4-268.4, *P*<.001 in all cases) were significantly higher for guided than for self-guided wb-CBT (Table S4 in Multimedia Appendix 2). However, this pattern reversed after 12 weeks, when guidance was no longer present, at which time both average minutes per week (0.4, SD 7.5 for self-guided vs 0.2, SD 4.4 for guided wb-CBT; *χ*^2^_1_=10.5, *P*=.001) and the proportions of people who spent ≥5, ≥10, and ≥30 minutes per week with the intervention (*χ*^2^_1_=5.3-17.2, *P*<.001 to *P*=.02) became significantly higher with self-guided than guided wb-CBT.

### Interactions Between Treatment Arm and Self-Guided wb-CBT Predicted Compliance

On the basis of the results regarding changes in patterns of observed compliance with self-guided versus guided wb-CBT after 12 weeks, we used the machine learning model described in the *Methods* section to create a prediction score for the extent to which each participant would be predicted to comply with self-guided wb-CBT between 13 and 52 weeks after randomization if randomly assigned to that arm. This predicted compliance score was then used as a specifier of the comparative intervention effects reported previously. It is noteworthy that 10-fold–cross-validated predicted compliance had a statistically significant but substantively weak association with observed compliance in the self-guided wb-CBT arm (*R*^2^=0.05; SE 0.02).

Exploratory analyses suggested that the best dichotomous distinction was between the 40% (528/1319) of participants with highest predicted compliance and the remaining 60% (791/1319) of participants. A significant interaction was found between this dichotomy and randomization to self-guided wb-CBT in predicting 12-month joint remission (*χ*^2^_1_=13.5, *P*<.001). In the 40% (528/1319) of participants with high predicted compliance, 12-month joint anxiety-depression remission rates varied significantly across arms (*χ*^2^_2_=17.5, *P*<.001) due to the remission rate being significantly higher with self-guided wb-CBT than with either guided wb-CBT (ARD=−9.3%; *χ*^2^_1_=7.3, *P*=.007) or TAU (ARD=11%; *χ*^2^_1_=19.3, *P*<.001), whereas there was no significant difference between guided wb-CBT and TAU (ARD=1.7%; *χ*^2^_1_=0.2, *P*=.62; Table 4). In the 60% (791/1319) of participants with low predicted compliance, in comparison, 12-month joint remission rates did not differ significantly across intervention arms (*χ*^2^_2_=1.2, *P*=.54).

**Table 4:** Average treatment effects across arms at 12 months with the adjusted risk differences (ARDs) for the joint remission outcome stratified by predicted compliance with self-guided web-based cognitive behavioral therapy (wb-CBT) in weeks 13 to 52.

		Total^a^			Guided^b^			Self-guided^c^			TAU^d,e^	
		Estimate^f^ (SE)	*P* value	Estimate (SE)		*P* value	Estimate (SE)		*P* value	Estimate (SE)		*P* value
**Joint remission on the GAD-7^g^ and PHQ-9^h,i^**												
	High–predicted compliance subsample^j^	38.4 (4.0)	<.001	35.8 (2.9)		<.001	45.1 (1.9)		<.001	34.1 (1.7)		<.001
	Low–predicted compliance subsample^k^	30.9 (4.0)	<.001	30.1 (3.0)		<.001	32.1 (2.7)		<.001	30.5 (1.6)		<.001

^a^High–predicted compliance subsample: n=528; low–predicted compliance subsample: n=791.

^b^High–predicted compliance subsample: n=175; low–predicted compliance subsample: n=270.

^c^High–predicted compliance subsample: n=195; low–predicted compliance subsample: n=244.

^d^High–predicted compliance subsample: n=158; low–predicted compliance subsample: n=277.

^e^TAU: treatment as usual.

^f^Estimate of joint remission rates on both the Generalized Anxiety Disorder–7 and Patient Health Questionnaire–9 or means of PHQ-ADS scores.

^g^GAD-7: Generalized Anxiety Disorder–7 (0-21).

^h^PHQ-9: Patient Health Questionnaire–9 (0-27).

^i^Joint remission was defined as scores of 0 to 4 on both the GAD-7 and PHQ-9.

^j^Guided versus self-guided wb-CBT: ARD=–9.3 (SE 3.4) and *P*=.006; TAU versus self-guided wb-CBT: ARD=–11.0 (SE 2.5) and *P*<.001; guided wb-CBT versus TAU: ARD=1.7 (SE 3.4) and *P*=.62; self-guided wb-CBT versus TAU: ARD=11.0 (SE 2.5) and *P*<.001. For the high–predicted compliance subsample, the 2-sided test with 2 *df* at the .05 level for the overall variations across the 3 arms on joint remission on the GAD-7 and PHQ-9 was statistically significant at 12 months (*χ*^2^_2_=17.5, *P*<.001). In addition, the overall variations across the 3 arms on mean PHQ-ADS scores were statistically significant at 12 months (*χ*^2^_2_=47.0, *P*<.001) but not at baseline (*χ*^2^_2_=0.3, *P*=.87) among participants with high predicted compliance.

^k^Guided versus self-guided wb-CBT: ARD=–1.9 (SE 4.0) and *P*=.64; TAU versus self-guided wb-CBT: ARD=–1.6 (SE 3.1) and *P*=.61; guided wb-CBT versus TAU: ARD=–0.4 (SE 3.4) and *P*=.91; self-guided wb-CBT versus TAU: ARD=1.6 (SE 3.1) and *P*=.61. For the low–predicted compliance subsample, the 2-sided test with 2 *df* at the .05 level for the overall variations across the 3 arms on joint remission on the GAD-7 and PHQ-9 was not statistically significant at 12 months (*χ*^2^_2_=1.2, *P*=.54). In addition, the overall variations across the 3 arms on mean PHQ-ADS scores were statistically significant at 12 months (*χ*^2^_2_=6.6, *P*=.04) but not at baseline (*χ*^2^_2_=0.6, *P*=.73) among participants with low predicted compliance.

A similar result was found for 12-month mean PHQ-ADS scores (Table 5), where scores varied significantly across intervention arms in the 40% (528/1319) of participants with high predicted compliance (*χ*^2^_2_=47.0, *P*<.001) due to a significantly lower mean for self-guided wb-CBT than for either guided wb-CBT (AMD=4.2; *χ*^2^_1_=26.2, *P*<.001) or TAU (AMD=−3.2; *χ*^2^_1_=30.1, *P*<.001) but with no significant difference between guided wb-CBT and TAU (AMD=1.0; *χ*^2^_1_=1.6, *P*=.20; Table 5). In the 60% (791/1319) of participants with low predicted compliance, in comparison, the mean PHQ-ADS score was significantly lower with self-guided wb-CBT than with TAU (AMD=−1.8; *χ*^2^_1_=5.9, *P*=.02), but mean PHQ-ADS scores did not differ significantly either between self-guided and guided wb-CBT (AMD=1.0; *χ*^2^_1_=1.2, *P*=.28) or between guided wb-CBT and TAU (AMD=−0.8; *χ*^2^_1_=0.6, *P*=.43).

**Table 5:** Average treatment effects across arms at 12 months with the adjusted mean differences (AMDs) for the Patient Health Questionnaire Anxiety and Depression Scale (PHQ-ADS) outcome stratified by predicted compliance with self-guided web-based cognitive behavioral therapy (wb-CBT) in weeks 13 to 52.

				Total^a^					Guided^b^					Self-guided^c^					TAU^d,e^		
				Estimate (SE)		*P* value		Estimate (SE)			*P* value		Estimate (SE)			*P* value		Estimate (SE)			*P* value
**Mean PHQ-ADS scores**																					
	**High–predicted compliance subsample^f^**																				
		Baseline	26.9 (2.7)		<.001		26.7 (1.5)			<.001		26.7 (1.5)			<.001		27.3 (1.6)			<.001	
		12 months	15.8 (0.9)		<.001		17.5 (0.7)			<.001		13.3 (0.5)			<.001		16.5 (0.4)			<.001	
	**Low–predicted compliance subsample^g^**																				
		Baseline	29.8 (2.3)		<.001		30.2 (1.4)			<.001		29.4 (1.4)			<.001		29.7 (1.2)			<.001	
		12 months	17.9 (1.1)		<.001		18.0 (0.8)			<.001		16.9 (0.5)			<.001		18.7 (0.5)			<.001	

^a^High–predicted compliance subsample: n=528; low–predicted compliance subsample: n=791.

^b^High–predicted compliance subsample: n=175; low–predicted compliance subsample: n=270.

^c^High–predicted compliance subsample: n=195; low–predicted compliance subsample: n=244.

^d^High–predicted compliance subsample: n=158; low–predicted compliance subsample: n=277.

^e^TAU: treatment as usual.

^f^Guided versus self-guided wb-CBT: AMD=4.2 (SE 0.8) and *P*<.001; TAU versus self-guided wb-CBT: AMD=3.2 (SE 0.6) and *P*<.001; guided wb-CBT versus TAU: AMD=1.0 (SE 0.8) and *P*=.21; self-guided wb-CBT versus TAU: AMD=–3.2 (SE 0.6) and *P*<.001. For the high–predicted compliance subsample, the 2-sided test with 2 *df* at the .05 level for the overall variations across the 3 arms on joint remission on the Generalized Anxiety Disorder–7 (GAD-7) and Patient Health Questionnaire–9 (PHQ-9) was statistically significant at 12 months (*χ*^2^_2_=17.5, *P*<.001). In addition, the overall variations across the 3 arms on mean PHQ-ADS scores were statistically significant at 12 months (*χ*^2^_2_=47.0, *P*<.001) but not at baseline (*χ*^2^_2_=0.3, *P*=.87) among participants with high predicted compliance.

^g^Guided versus self-guided wb-CBT: AMD=1.0 (SE 1.0) and *P*=.32; TAU versus self-guided wb-CBT: AMD=1.8 (SE 0.7) and *P*=.01; guided wb-CBT versus TAU: AMD=–0.8 (SE 1.0) and *P*=.42; self-guided wb-CBT versus TAU: AMD=–1.8 (SE 0.7) and *P*=.01. For the low–predicted compliance subsample, the 2-sided test with 2 *df* at the .05 level for the overall variations across the 3 arms on joint remission on the GAD-7 and PHQ-9 was not statistically significant at 12 months (*χ*^2^_2_=1.2, *P*=.54). In addition, the overall variations across the 3 arms on mean PHQ-ADS scores were statistically significant at 12 months (*χ*^2^_2_=6.6, *P*=.04) but not at baseline (*χ*^2^_2_=0.6, *P*=.73) among participants with low predicted compliance.

It is noteworthy that heterogeneity was quite different for 3-month outcomes, where we showed in a previous report that the aggregate joint remission rate was higher for guided wb-CBT than for the other arms [16]. Our new analyses reported in this paper for the first time found significant variation in these comparative effects as a function of the same aforementioned predicted compliance measure (ie, predicted compliance with self-guided wb-CBT over weeks 13 to 52 in the high–predicted compliance subgroup; *χ*^2^_2_=9.5, *P*=.009; Table S5 in Multimedia Appendix 2). However, in the 40% (528/1319) of participants with high predicted compliance, this 3-month joint remission rate remained significantly higher for guided wb-CBT than for either self-guided wb-CBT (ARD=18.3%; *χ*^2^_1_=8.4, *P*=.004) or TAU (ARD=15.1%; *χ*^2^_1_=5.6, *P*=.02) and with no significant difference between self-guided wb-CBT and TAU (ARD=−3.3%; *χ*^2^_1_=0.3, *P*=.60). In the 60% (791/1319) of participants with low predicted compliance, in comparison, 3-month joint remission rates did not vary significantly across arms (*χ*^2^_2_=3.6, *P*=.16). In addition, 3-month PHQ-ADS means did not vary significantly across arms either in the 40% (528/1319) of high predicted compliers (*χ*^2^_2_=4.2, *P*=.12) or the 60% (791/1319) of low predicted compliers (*χ*^2^_2_=3.4, *P*=.18; Table S5 in Multimedia Appendix 2).

### The Baseline Covariates That Most Strongly Predicted Compliance With Self-Guided wb-CBT

**Figure 2:**
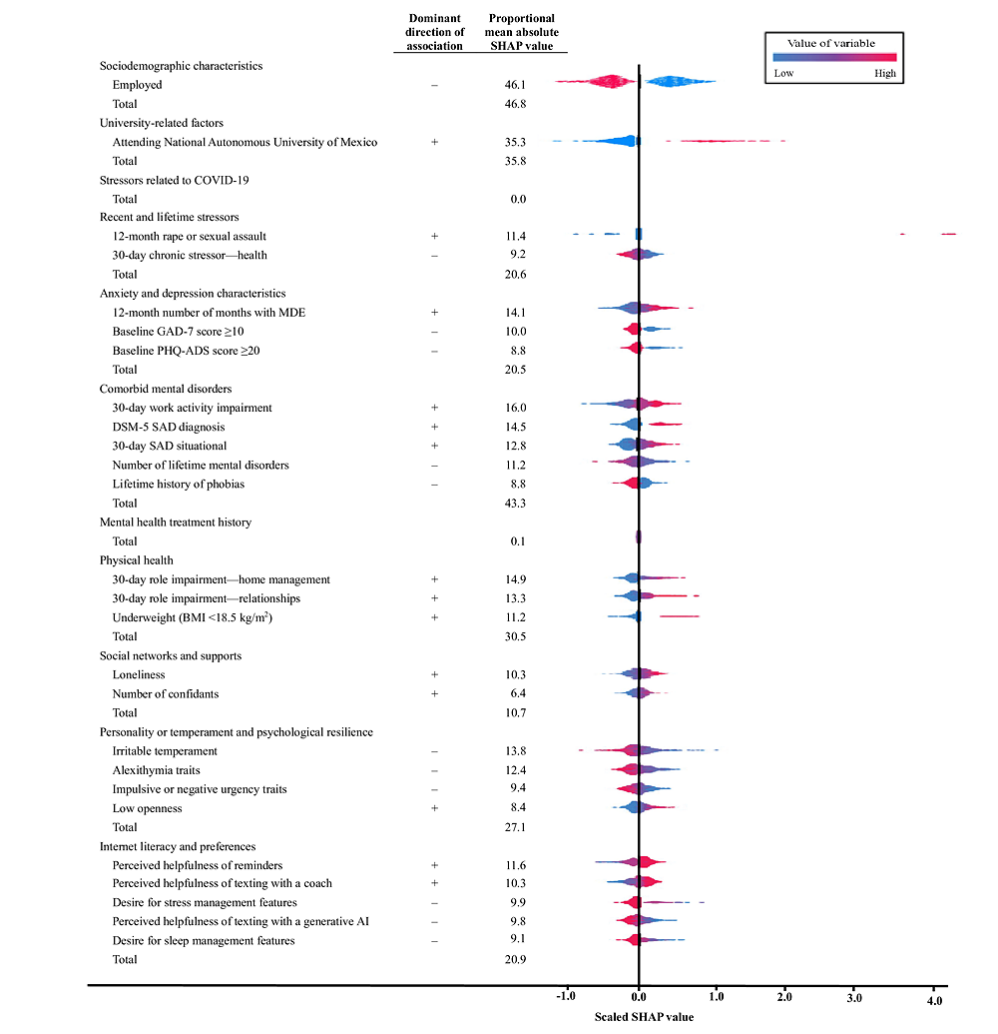
Predictors of projected compliance in the self-guided web-based cognitive behavioral therapy (wb-CBT) arm (n=439). Compliance is measured as minutes spent on the wb-CBT intervention. “Dominant direction of association” refers to the Shapley additive explanations (SHAP) values. “Proportional mean absolute SHAP value” refers to the key predictors, which are those within each domain that rank among the top 5 with mean absolute SHAP values of ≥0.01. AI: artificial intelligence; DSM-5: Diagnostic and Statistical Manual of Mental Disorders, Fifth Edition; GAD-7: Generalized Anxiety Disorder–7; MDE: major depressive episode; PHQ-ADS: Patient Health Questionnaire Anxiety and Depression Scale; SAD: social anxiety disorder.

Given the significant heterogeneity in intervention effects associated with predicted compliance, an exploration of the baseline variables defining this construct is warranted. The strongest baseline predictors were not being employed (SHAP_P_=46.1%), comorbid mental disorders (SHAP_P_=43.3%), attending National Autonomous University of Mexico (SHAP_P_=35.3%), and physical health (SHAP_P_=30.5%; Figure 2). In Figure 2, *Dominant direction of association* refers to the SHAP values, which may vary for a particular predictor among participants with identical scores on that predictor as they interact with other predictors, resulting in fluctuations in the association’s sign between the predictor and the outcome. The predominant association direction was determined through visual examination of the beeswarm plot displayed on the right side of the figure. *Employed* refers to students presently working for any number of hours. *Baseline PHQ-ADS score of ≥20* refers to participants with baseline PHQ-ADS scores of ≥20 but without scores of 0 or 1 on the first 2 items of the PHQ-9 and with baseline PHQ-9 scores of ≥10. *30-day work activity impairment* refers to having worked less carefully during the previous 30 days due to mental health problems. *30-day SAD situational* refers to social situation–related anxiety (intensity of anxiety or fear × frequency of anxiety). *Lifetime history of phobias* refers to a self-reported history of extreme fears of specific objects or situations. *30-day role impairment—home management* and *30-day role impairment—relationships* refer to how much physical health problems interfered with their life domain (scale of 0-10). *Loneliness* refers to frequency × severity of loneliness (scale of 0-16). *Perceived helpfulness of reminders* and *Perceived helpfulness of texting with a generative AI* refer to higher scores that indicate greater perceived helpfulness of the specific wb-CBT feature (scale of 0-3). *Desire for stress management features* and *Desire for sleep management features* refer to higher scores that indicate greater perceived importance of the specific wb-CBT feature (scale of 0-3).

For comorbid mental disorders, the most important predictors of high compliance were high work impairment due to mental disorders, comorbid social anxiety disorder (SAD), high situational SAD severity, low lifetime comorbidity, and absence of lifetime history of extreme fears of specific objects or situations. For physical health, the most important predictors were role impairment in home management, role impairment in relationship areas, and underweight status. For personality factors, the most important predictors were low irritability, alexithymia, impulsivity or negative urgency, and openness. For internet preferences, the most important predictors were high perceived helpfulness of reminders and texting with a coach and lower desire for stress and sleep management features. For stressors, the most significant predictors were past-year sexual assault and lower past-month chronic health stressors. Other important predictors included higher past-year number of months with major depressive episodes, absence of high baseline GAD-7 and PHQ-ADS scores, higher perceived loneliness, and higher number of confidants.

## Discussion

### Principal Findings

We focused on university students with anxiety or depression in 2 upper-middle–income Latin American countries, Colombia and Mexico. There are 2 main findings that are noteworthy. First, 12-month outcomes were significantly better for participants randomly assigned to self-guided wb-CBT than for those randomly assigned to either guided wb-CBT or TAU, whereas 12-month outcomes were not better for those randomly assigned to guided wb-CBT than for those randomly assigned to TAU, even though 3-month outcomes were best for those randomly assigned to guided wb-CBT. Second, we found that these significant differences were confined to the 40% (528/1319) of participants with the highest predicted compliance with self-guided wb-CBT over weeks 13 to 52 based on the covariates assessed before randomization. This specification suggests that the superiority of self-guided wb-CBT at 12 months is due to the higher continued use of self-guided wb-CBT than of guided wb-CBT after the guidance ends.

### Comparison With Previous Studies

The only previously published systematic review and meta-analysis we are aware of that compared the effects of guided and self-guided wb-CBT over longer periods found that the superiority of guided wb-CBT in treating depression at 3 months was no longer present at 6 or 12 months [12]. That review did not address anxiety or comorbid depression and anxiety. Our recent meta-analysis [18], in comparison, which looked at the comparative effects of guided and self-guided wb-CBT on all common mental disorders, found no consistent differences either in remission rates or in mean symptom reduction between guided and self-guided wb-CBT at ≥12 months.

The small number of previous wb-CBT studies that examined associations of compliance with outcomes in standard per-protocol or as-treated analyses yielded inconsistent results on whether compliance is a significant correlate of outcomes [31,46]. Previous IV studies, in comparison, have consistently found that estimates of treatment effects in the subsample of patients who comply with treatment only because of randomization (ie, excluding from consideration the subset of patients who would obtain the treatment even if randomly assigned to the control group) are stronger than intervention effects in total-sample estimates [39,47,48]. However, this result is trivially true because these IV estimates were the equivalent of aggregate ARD and AMD estimates divided by the proportion of participants who complied with the intervention [49], which means that the IV estimates are definitionally at least as large as the total-sample estimates. However, these IV estimates were biased to the extent that they failed to capture the full effects of the interventions using dichotomous characterizations of compliance.

We are aware of no studies other than ours that have examined the effect of compliance by using baseline covariates to create a measure of predicted compliance. A great appeal of this approach is that it yields valid estimates of heterogeneity in intervention effects with respect to baseline variables that do not rely on the implausible assumptions needed in conventional per-protocol, as-treated, and IV analyses. Another appeal is that our approach provides a principled basis for specifying a precision treatment rule that can be used to help guide allocation of interventions to future patients in a way that maximizes the scalability of treatment.

### Clinical Implications

There are several clinical implications to these findings. First, the results argue that the dominant view of self-guided wb-CBT as inferior to guided wb-CBT is unwarranted if one considers 12-month outcomes. Ideally, we would want to promote long-term remission rather than the shorter-term remission that has been the focus of most previous wb-CBT research. Second, the importance of sustained use of the self-guided wb-CBT platform suggests that learning CBT skills is not enough in itself but that ongoing practice and review of materials is needed, presumably to help consolidate acquired CBT skills in various life contexts. Participants randomly assigned to guided wb-CBT were trained to be extrinsically, instead of intrinsically, motivated to engage with the intervention by virtue of the external reinforcement they received from their guide, leading to a greater reduction in use of the intervention once guidance ended. Importantly, this was true even for the subset of these participants whose baseline profiles suggested that they would have complied intrinsically if they had been randomly assigned to self-guided wb-CBT. The individuals with this baseline profile who were randomly assigned to self-guided wb-CBT, in comparison, had significantly better longer-term outcomes than if they had been randomly assigned to guided wb-CBT because self-guidance allowed these individuals to consolidate their intrinsic motivation to support longer-term use of the intervention. One implication of this finding is that guided wb-CBT programs need to consider how best to foster intrinsic motivation, possibly through strategies such as intermittent guidance, the use of longer-term booster sessions, tapering guidance over a longer time, or offering guidance on demand or just-in-time adaptive guidance. Although some limited research on such possibilities exists [50,51], our results suggest that this area warrants further study.

For individuals assigned to the self-guided modality, compliance may have been more intrinsically motivated from day 1 as these participants never had the positive reinforcement of a human guide, increasing the probability that participants with high predicted compliance continued to use the intervention over the full 12-month access period. Interestingly, not being employed and having high comorbidity (especially SAD) were among the strongest predictors of longer-term self-guided wb-CBT compliance. Why not being employed would be a predictor is unclear considering that this was a university student sample, but it might be due to having more time available and being less overextended. Participants with SAD might have complied more with the self-guided wb-CBT because they found it less threatening than interacting with a guide.

Finally, whether the focus of treatment planning should be more on short-term or longer-term outcomes is unclear. It may be more critical to reduce symptomatology as quickly as possible (and, thus, prioritize short-term outcomes), but we also want intervention effects to persist because of the recurrent nature of anxiety and depression. It is unclear whether an approach exists to do both given that guidance appears to increase short-term compliance but reduce longer-term compliance.

### Limitations

Our study had 4 noteworthy limitations. First, this study was carried out during the COVID-19 pandemic. It is unclear whether this influenced the results. Second, overall intervention compliance was low. This is consistent with many other web-based intervention studies [14], may be due not only to characteristics of the users but also to characteristics of the programs [52], and might have been exacerbated by the pandemic. Because of this low compliance and context, our results involving interactions between intervention assignment and predicted compliance might not be generalizable beyond the specific web-based interventions considered here. Third, even though we had a large set of baseline covariates, these variables were chosen as predictors of treatment response rather than of intervention compliance. Future research designed to study the effects of compliance should include a broader set of baseline covariates that include known predictors of compliance [53-55]. Fourth, TAU was heterogeneous across universities and was mainly administered via videoconferencing because this trial was conducted during the COVID-19 pandemic lockdown. Taken together, these limitations suggest that caution should be taken in assuming that the results are generalizable beyond the specific time and setting in which this trial was carried out.

### Conclusions

Despite these limitations, this study had several strengths and raises several important clinical considerations. This was a comparatively large clinical trial in 2 Latin American LMICs using a culturally adapted wb-CBT program in which the guided and self-guided modalities were identical in content apart from the guided feedback. Our results question the generally accepted notion that guided wb-CBT is superior to self-guided wb-CBT. Indeed, we found that the self-guided modality was superior to guided wb-CBT over a 12-month follow-up period. Importantly, we were able to explain this advantage using a measure of predicted longer-term compliance with self-guided wb-CBT based on predictor information available before randomization. This measure could be used in the future to help guide intervention assignment. It would doubtlessly be possible to improve this predicted compliance score by expanding the baseline assessment in the future. Whether we should offer self-guided wb-CBT to all participants with high predicted self-guided compliance is a question worth considering, although it would be wise to confirm the stability of this specification in the next iteration of our trial before doing so. In addition, we need to consider what interventions to offer students with lower predicted compliance. It would be useful to focus trials of distinct interventions on that segment of the population.

## Data Availability

The deidentified datasets generated or analyzed during this study are available to qualified researchers in the National Institute of Mental Health Data Archive [56].

## Appendix

Multimedia Appendix 1 CONSORT-eHEALTH checklist (V 1.6.1).

Multimedia Appendix 2 Supplementary analyses of baseline participant characteristics, interactions predicting self-administered questionnaire completion, follow-up rates, and average minutes spent on the web-based cognitive behavioral therapy intervention per treatment arm.

## Abbreviations

AMD: adjusted mean difference
ARD: adjusted risk difference
ATE: average treatment effect
CBT: cognitive behavioral therapy
CONSORT: Consolidated Standards of Reporting Trials
GAD-7: Generalized Anxiety Disorder–7
IV: instrumental variable
LMIC: low- or middle-income country
PHQ-9: Patient Health Questionnaire–9
PHQ-ADS: Patient Health Questionnaire Anxiety and Depression Scale
SAD: social anxiety disorder
SAQ: self-administered questionnaire
SHAP: Shapley additive explanations
TAU: treatment as usual
wb-CBT: web-based cognitive behavioral therapy
