# Supplementary material for: The Effect of Predicted Compliance With a Web-Based Intervention for Anxiety and Depression Among Latin American University Students: Randomized Controlled Trial
Source: JMIR Ment Health. 2025 Feb 28;12:e64251. doi: 10.2196/64251 (PMC11909483; doi:10.2196/64251)
Supplement: Multimedia Appendix 1 [file mental_v12i1e64251_app1.pdf]

CONSORT-EHEALTH (V 1.6.1) - Submission/Publication Form

The CONSORT-EHEALTH checklist is intended for authors of randomized trials evaluating web-based and Internet-based applications/interventions, including mobile interventions, electronic games (incl multiplayer games), social media, certain telehealth applications, and other interactive and/or networked electronic applications. Some of the items (e.g. all subitems under item 5 - description of the intervention) may also be applicable for other study designs.

The goal of the CONSORT EHEALTH checklist and guideline is to be
a) a guide for reporting for authors of RCTs,
b) to form a basis for appraisal of an ehealth trial (in terms of validity)

CONSORT-EHEALTH items/subitems are MANDATORY reporting items for studies published in the Journal of Medical Internet Research and other journals / scientific societies endorsing the checklist.

Items numbered 1., 2., 3., 4a., 4b etc are original CONSORT or CONSORT-NPT (non-pharmacologic treatment) items.
Items with Roman numerals (i., ii, iii, iv etc.) are CONSORT-EHEALTH extensions/ clarifications.

As the CONSORT-EHEALTH checklist is still considered in a formative stage, we would ask that you also RATE ON A SCALE OF 1-5 how important/useful you feel each item is FOR THE PURPOSE OF THE CHECKLIST and reporting guideline (optional).

Mandatory reporting items are marked with a red *.
In the textboxes, either copy & paste the relevant sections from your manuscript into this form - please include any quotes from your manuscript in QUOTATION MARKS,
or answer directly by providing additional information not in the manuscript, or elaborating on why the item was not relevant for this study.

YOUR ANSWERS WILL BE PUBLISHED AS A SUPPLEMENTARY FILE TO YOUR PUBLICATION IN JMIR AND ARE CONSIDERED PART OF YOUR PUBLICATION (IF ACCEPTED).
Please fill in these questions diligently. Information will not be copyedited, so please use proper spelling and grammar, use correct capitalization, and avoid abbreviations.

DO NOT FORGET TO SAVE AS PDF _AND_ CLICK THE SUBMIT BUTTON SO YOUR ANSWERS ARE IN OUR DATABASE !!!

Citation Suggestion (if you append the pdf as Appendix we suggest to cite this paper in the caption):

Eysenbach G, CONSORT-EHEALTH Group
CONSORT-EHEALTH: Improving and Standardizing Evaluation Reports of Web-based and Mobile Health Interventions
J Med Internet Res 2011;13(4):e126
URL: http://www.jmir.org/2011/4/e126/
doi: 10.2196/jmir.1923
PMID: 22209829

willett@hcp.med.harvard.edu Switch account

Not shared

Draft saved

* Indicates required question

Your name *

First Last

Corina Benjet

Primary Affiliation (short), City, Country *

University of Toronto, Toronto, Canada

National Institute of Psychiatry Ramón de la Fu

Your e-mail address *

abc@gmail.com

cbenjet@gmail.com

Title of your manuscript *

Provide the (draft) title of your manuscript.

The Effect of Predicted Intervention Compliance on a Web-based Intervention for Anxiety and Depression Among Latin American University Students: A Randomized Controlled Trial

Name of your App/Software/Intervention *

If there is a short and a long/alternate name, write the short name first and add the long name in brackets.

SilverCloud Health by Amwell's Space from An

Evaluated Version (if any)

e.g. "V1", "Release 2017-03-01", "Version 2.0.27913"

Culturally adapted Latin American version Yo P

Language(s) *

What language is the intervention/app in? If multiple languages are available, separate by comma (e.g. "English, French")

Spanish

URL of your Intervention Website or App

e.g. a direct link to the mobile app on app in appstore (itunes, Google Play), or URL of the website. If the intervention is a DVD or hardware, you can also link to an Amazon page.

https://yopuedosentirmebien.silvercloudhealth.com/

URL of an image/screenshot (optional)

Your answer

Accessibility *

Can an enduser access the intervention presently?

- ○ access is free and open
- ○ access only for special usergroups, not open
- ○ access is open to everyone, but requires payment/subscription/in-app purchases
- ○ app/intervention no longer accessible
- ◉ Other: Available for research purposes only

Primary Medical Indication/Disease/Condition *

e.g. "Stress", "Diabetes", or define the target group in brackets after the condition, e.g. "Autism (Parents of children with)", "Alzheimers (Informal Caregivers of)"

Anxiety and/or Depression

Primary Outcomes measured in trial *

comma-separated list of primary outcomes reported in the trial

Anxiety/depression remission (GAD-7=0-4 and

Secondary/other outcomes

Are there any other outcomes the intervention is expected to affect?

Anxiety/depression symptom remission

Recommended "Dose" *

What do the instructions for users say on how often the app should be used?

- ○ Approximately Daily
- ◉ Approximately Weekly
- ○ Approximately Monthly
- ○ Approximately Yearly
- ○ "as needed"
- ○ Other:

Approx. Percentage of Users (starters) still using the app as recommended after 3 months *

- ( ) unknown / not evaluated
- ( ) 0-10%
- (x) 11-20%
- ( ) 21-30%
- ( ) 31-40%
- ( ) 41-50%
- ( ) 51-60%
- ( ) 61-70%
- ( ) 71%-80%
- ( ) 81-90%
- ( ) 91-100%
- ( ) Other:

Overall, was the app/intervention effective? *

- (selected) yes: all primary outcomes were significantly better in intervention group vs control
- partly: SOME primary outcomes were significantly better in intervention group vs control
- no statistically significant difference between control and intervention
- potentially harmful: control was significantly better than intervention in one or more outcomes
- inconclusive: more research is needed
- Other:

Article Preparation Status/Stage *

At which stage in your article preparation are you currently (at the time you fill in this form)

- not submitted yet - in early draft status
- not submitted yet - in late draft status, just before submission
- submitted to a journal but not reviewed yet
- (selected) submitted to a journal and after receiving initial reviewer comments
- submitted to a journal and accepted, but not published yet
- published
- Other:

Journal *

If you already know where you will submit this paper (or if it is already submitted), please provide the journal name (if it is not JMIR, provide the journal name under "other")

- ○ not submitted yet / unclear where I will submit this
- ○ Journal of Medical Internet Research (JMIR)
- ○ JMIR mHealth and UHealth
- ○ JMIR Serious Games
- ◉ JMIR Mental Health
- ○ JMIR Public Health
- ○ JMIR Formative Research
- ○ Other JMIR sister journal
- ○ Other:

Is this a full powered effectiveness trial or a pilot/feasibility trial? *

- ○ Pilot/feasibility
- ◉ Fully powered

Manuscript tracking number *

If this is a JMIR submission, please provide the manuscript tracking number under "other" (The ms tracking number can be found in the submission acknowledgement email, or when you login as author in JMIR. If the paper is already published in JMIR, then the ms tracking number is the four-digit number at the end of the DOI, to be found at the bottom of each published article in JMIR)

- ○ no ms number (yet) / not (yet) submitted to / published in JMIR
- ◉ Other: ms #64251

TITLE AND ABSTRACT

1a) TITLE: Identification as a randomized trial in the title

1a) Does your paper address CONSORT item 1a? *

I.e does the title contain the phrase "Randomized Controlled Trial"? (if not, explain the reason under "other")

- ◉ yes
- ○ Other:

1a-i) Identify the mode of delivery in the title

Identify the mode of delivery. Preferably use “web-based” and/or “mobile” and/or “electronic game” in the title. Avoid ambiguous terms like “online”, “virtual”, “interactive”. Use “Internet-based” only if Intervention includes non-web-based Internet components (e.g. email), use “computer-based” or “electronic” only if offline products are used. Use “virtual” only in the context of “virtual reality” (3-D worlds). Use “online” only in the context of “online support groups”. Complement or substitute product names with broader terms for the class of products (such as “mobile” or “smart phone” instead of “iphone”), especially if the application runs on different platforms.

	1	2	3	4	5	
subitem not at all important	○	○	○	○	◉	essential

Clear selection

Does your paper address subitem 1a-i? *

Copy and paste relevant sections from manuscript title (include quotes in quotation marks "like this" to indicate direct quotes from your manuscript), or elaborate on this item by providing additional information not in the ms, or briefly explain why the item is not applicable/relevant for your study

"Web-based Intervention"

1a-ii) Non-web-based components or important co-interventions in title

Mention non-web-based components or important co-interventions in title, if any (e.g., “with telephone support”).

	1	2	3	4	5	
subitem not at all important	◉	○	○	○	○	essential

Clear selection

Does your paper address subitem 1a-ii?

Copy and paste relevant sections from manuscript title (include quotes in quotation marks "like this" to indicate direct quotes from your manuscript), or elaborate on this item by providing additional information not in the ms, or briefly explain why the item is not applicable/relevant for your study

There were no non-web based components.

1a-iii) Primary condition or target group in the title

Mention primary condition or target group in the title, if any (e.g., “for children with Type I Diabetes”) Example: A Web-based and Mobile Intervention with Telephone Support for Children with Type I Diabetes: Randomized Controlled Trial

	1	2	3	4	5	
subitem not at all important	○	○	○	○	◉	essential

Clear selection

Does your paper address subitem 1a-iii? *

Copy and paste relevant sections from manuscript title (include quotes in quotation marks "like this" to indicate direct quotes from your manuscript), or elaborate on this item by providing additional information not in the ms, or briefly explain why the item is not applicable/relevant for your study

“for anxiety and depression among Latin American University Students”

1b) ABSTRACT: Structured summary of trial design, methods, results, and conclusions

NPT extension: Description of experimental treatment, comparator, care providers, centers, and blinding status.

1b-i) Key features/functionalities/components of the intervention and comparator in the METHODS section of the ABSTRACT

Mention key features/functionalities/components of the intervention and comparator in the abstract. If possible, also mention theories and principles used for designing the site. Keep in mind the needs of systematic reviewers and indexers by including important synonyms. (Note: Only report in the abstract what the main paper is reporting. If this information is missing from the main body of text, consider adding it)

	1	2	3	4	5	
subitem not at all important	○	○	○	◉	○	essential

Clear selection

Does your paper address subitem 1b-i? *

Copy and paste relevant sections from the manuscript abstract (include quotes in quotation marks "like this" to indicate direct quotes from your manuscript), or elaborate on this item by providing additional information not in the ms, or briefly explain why the item is not applicable/relevant for your study

“guided wb-CBT with weekly asynchronous written human feedback, self-guided wb-CBT with the same content as the guided modality

1b-ii) Level of human involvement in the METHODS section of the ABSTRACT

Clarify the level of human involvement in the abstract, e.g., use phrases like “fully automated” vs. “therapist/nurse/care provider/physician-assisted” (mention number and expertise of providers involved, if any). (Note: Only report in the abstract what the main paper is reporting. If this information is missing from the main body of text, consider adding it)

	1	2	3	4	5	
subitem not at all important	○	○	○	◉	○	essential

Clear selection

Does your paper address subitem 1b-ii?

Copy and paste relevant sections from the manuscript abstract (include quotes in quotation marks "like this" to indicate direct quotes from your manuscript), or elaborate on this item by providing additional information not in the ms, or briefly explain why the item is not applicable/relevant for your study

“guided wb-CBT with weekly asynchronous written human feedback, self-guided wb-CBT with the same content as the guided modality and TAU as provided at each university.”

1b-iii) Open vs. closed, web-based (self-assessment) vs. face-to-face assessments in the METHODS section of the ABSTRACT

Mention how participants were recruited (online vs. offline), e.g., from an open access website or from a clinic or a closed online user group (closed usergroup trial), and clarify if this was a purely web-based trial, or there were face-to-face components (as part of the intervention or for assessment). Clearly say if outcomes were self-assessed through questionnaires (as common in web-based trials). Note: In traditional offline trials, an open trial (open-label trial) is a type of clinical trial in which both the researchers and participants know which treatment is being administered. To avoid confusion, use “blinded” or “unblinded” to indicated the level of blinding instead of “open”, as “open” in web-based trials usually refers to “open access” (i.e. participants can self-enrol). (Note: Only report in the abstract what the main paper is reporting. If this information is missing from the main body of text, consider adding it)

	1	2	3	4	5	
subitem not at all important	○	○	◉	○	○	essential

Clear selection

Does your paper address subitem 1b-iii?

Copy and paste relevant sections from the manuscript abstract (include quotes in quotation marks "like this" to indicate direct quotes from your manuscript), or elaborate on this item by providing additional information not in the ms, or briefly explain why the item is not applicable/relevant for your study

"recruited either through email and social media outreach invitations or from waiting lists of campus mental health clinics"

1b-iv) RESULTS section in abstract must contain use data

Report number of participants enrolled/assessed in each group, the use/uptake of the intervention (e.g., attrition/adherence metrics, use over time, number of logins etc.), in addition to primary/secondary outcomes. (Note: Only report in the abstract what the main paper is reporting. If this information is missing from the main body of text, consider adding it)

	1	2	3	4	5	
subitem not at all important	○	○	○	○	◉	essential

Clear selection

Does your paper address subitem 1b-iv?

Copy and paste relevant sections from the manuscript abstract (include quotes in quotation marks "like this" to indicate direct quotes from your manuscript), or elaborate on this item by providing additional information not in the ms, or briefly explain why the item is not applicable/relevant for your study

“Participants randomized to the guided wb-CBT arm spent twice as many minutes logged on as those in the self-guided wb-CBT arm in the first 12 weeks (12.4 vs. 5.9; χ21 = 107.1, P < .001) whereas participants in the self-guided wb-CBT arm spent twice as many minutes logged on as those in the guided wb-CBT arm in weeks 13-52 (0.4 vs. 0.2; χ21 = 10.5, P=.001). Subgroup analysis showed that this longer-term superiority of self-guided wb-CBT was confined to the roughly 40% of participants with high predicted self-guided wb-CBT compliance beyond 3 months based on a counter-factual nested cross-validated machine leaning model. 12-month outcome differences were nonsignificant across arms among other participants.”

1b-v) CONCLUSIONS/DISCUSSION in abstract for negative trials

Conclusions/Discussions in abstract for negative trials: Discuss the primary outcome - if the trial is negative (primary outcome not changed), and the intervention was not used, discuss whether negative results are attributable to lack of uptake and discuss reasons. (Note: Only report in the abstract what the main paper is reporting. If this information is missing from the main body of text, consider adding it)

	1	2	3	4	5	
subitem not at all important	○	○	○	○	◉	essential

Clear selection

Does your paper address subitem 1b-v?

Copy and paste relevant sections from the manuscript abstract (include quotes in quotation marks "like this" to indicate direct quotes from your manuscript), or elaborate on this item by providing additional information not in the ms, or briefly explain why the item is not applicable/relevant for your study

We mention that negative results at 12 months are confined to those with low predicted compliance.

INTRODUCTION

2a) In INTRODUCTION: Scientific background and explanation of rationale

2a-i) Problem and the type of system/solution

Describe the problem and the type of system/solution that is object of the study: intended as stand-alone intervention vs. incorporated in broader health care program? Intended for a particular patient population? Goals of the intervention, e.g., being more cost-effective to other interventions, replace or complement other solutions? (Note: Details about the intervention are provided in “Methods” under 5)

	1	2	3	4	5	
subitem not at all important	○	○	◉	○	○	essential

Clear selection

Does your paper address subitem 2a-i? *

Copy and paste relevant sections from the manuscript (include quotes in quotation marks "like this" to indicate direct quotes from your manuscript), or elaborate on this item by providing additional information not in the ms, or briefly explain why the item is not applicable/relevant for your study

"The current report presents the first results of a controlled trial in LMICs to compare the effects of guided and self-guided wb-CBT 12 months after randomization. We focus on our sample of Colombian and Mexican university students having anxiety or depression. We also expand our earlier preplanned investigation of heterogeneity in comparative intervention effects with a secondary analysis focused on determining whether a subset of participants can be identified at baseline (i.e., prior to randomization) who would have equal or better longer-term outcomes with self-guided compared to guided wb-CBT and the extent to which differential long-term intervention compliance might account for such heterogeneity. "

2a-ii) Scientific background, rationale: What is known about the (type of) system

Scientific background, rationale: What is known about the (type of) system that is the object of the study (be sure to discuss the use of similar systems for other conditions/ diagnoses, if appropiate), motivation for the study, i.e. what are the reasons for and what is the context for this specific study, from which stakeholder viewpoint is the study performed, potential impact of findings [2]. Briefly justify the choice of the comparator.

	1	2	3	4	5	
subitem not at all important	○	○	◉	○	○	essential

Clear selection

Does your paper address subitem 2a-ii? *

Copy and paste relevant sections from the manuscript (include quotes in quotation marks "like this" to indicate direct quotes from your manuscript), or elaborate on this item by providing additional information not in the ms, or briefly explain why the item is not applicable/relevant for your study

“Guided modalities are generally found to be more effective than self-guided modalities [12], although a recent meta-analysis suggests that this might be less true in low and middle-income countries due to self-guided wb-CBT having effect sizes equally as high as those of guided wb-CBT (LMIC) [13]. Treatment compliance is generally higher in guided than self-guided modalities and is considered the main reason guided wb-CBT usually yields better outcomes than self-guided wb-CBT [14]. Broad interest exists in improving uptake and effectiveness of self-guided wb-CBT given its much lower cost and great potential for scalability, reach, and affordability. In addition, there is interest in determining whether some subset of participants might be helped as much or more by self-guided as guided wb-CBT and, if so, developing a precision treatment rule that targets assignment of the different modalities in a way that maximizes clinical benefit at the lowest possible cost [15]… A limitation of our work so far, though, is that only short-term effects (i.e., 3-months post-randomization) have been examined. This limitation is shared by the larger wb-CBT literature, which tells us much less about longer-term than short-term effects. In a recent meta-analysis of all randomized controlled trials evaluating the comparative effects of guided and self-guided wb-CBT on common mental disorders over 12 months or longer, we found no consistent difference in the longer-term effects of guided compared to self-guided wb-CBT although both modalities continued to be associated with significantly better outcomes than controls [18]. Importantly, though, none of the studies in this meta-analysis came from low- or middle-income countries (LMIC).“

2b) In INTRODUCTION: Specific objectives or hypotheses

Does your paper address CONSORT subitem 2b? *

Copy and paste relevant sections from the manuscript (include quotes in quotation marks "like this" to indicate direct quotes from your manuscript), or elaborate on this item by providing additional information not in the ms, or briefly explain why the item is not applicable/relevant for your study

“to compare the effects of guided and self-guided wb-CBT 12 months after randomization… with a secondary analysis focused on determining whether a subset of participants can be identified at baseline (i.e., prior to randomization) who would have equal or better longer-term outcomes with self-guided compared to guided wb-CBT and the extent to which differential long-term intervention compliance might account for such heterogeneity.”

METHODS

3a) Description of trial design (such as parallel, factorial) including allocation ratio

Does your paper address CONSORT subitem 3a? *

Copy and paste relevant sections from the manuscript (include quotes in quotation marks "like this" to indicate direct quotes from your manuscript), or elaborate on this item by providing additional information not in the ms, or briefly explain why the item is not applicable/relevant for your study

This is a multisite, assessor-blinded randomized clinical trial. Participants were block randomized, with stratification by sex and severity of baseline anxiety and depression and equal allocation across the 3 intervention arms.

3b) Important changes to methods after trial commencement (such as eligibility criteria), with reasons

Does your paper address CONSORT subitem 3b? *

Copy and paste relevant sections from the manuscript (include quotes in quotation marks "like this" to indicate direct quotes from your manuscript), or elaborate on this item by providing additional information not in the ms, or briefly explain why the item is not applicable/relevant for your study

Not applicable.

3b-i) Bug fixes, Downtimes, Content Changes

Bug fixes, Downtimes, Content Changes: ehealth systems are often dynamic systems. A description of changes to methods therefore also includes important changes made on the intervention or comparator during the trial (e.g., major bug fixes or changes in the functionality or content) (5-iii) and other “unexpected events” that may have influenced study design such as staff changes, system failures/downtimes, etc. [2].

	1	2	3	4	5	
subitem not at all important	(•)	( )	( )	( )	( )	essential

Clear selection

Does your paper address subitem 3b-i?

Copy and paste relevant sections from the manuscript (include quotes in quotation marks "like this" to indicate direct quotes from your manuscript), or elaborate on this item by providing additional information not in the ms, or briefly explain why the item is not applicable/relevant for your study

Not applicable.

4a) Eligibility criteria for participants

Does your paper address CONSORT subitem 4a? *

Copy and paste relevant sections from the manuscript (include quotes in quotation marks "like this" to indicate direct quotes from your manuscript), or elaborate on this item by providing additional information not in the ms, or briefly explain why the item is not applicable/relevant for your study

“Inclusion criteria were completing the baseline assessment and reporting clinically significant anxiety (Generalized Anxiety Disorder [GAD-7] scores of 10 or above) [19] and/or depression (Patient Health Questionnaire [PHQ-9] scores of 10 or above) [20] and consenting to be randomized to either guided wb-CBT, self-guided wb-CBT or TAU. Exclusion criteria were reporting recent suicidal ideation with intent or screening positive for a history of bipolar disorder or nonaffective psychosis.”

4a-i) Computer / Internet literacy

Computer / Internet literacy is often an implicit “de facto” eligibility criterion - this should be explicitly clarified.

	1	2	3	4	5	
subitem not at all important	○	◉	○	○	○	essential

Clear selection

Does your paper address subitem 4a-i?

Copy and paste relevant sections from the manuscript (include quotes in quotation marks "like this" to indicate direct quotes from your manuscript), or elaborate on this item by providing additional information not in the ms, or briefly explain why the item is not applicable/relevant for your study

Because the participants are university students, they are considered to have computer/ internet literacy.

4a-ii) Open vs. closed, web-based vs. face-to-face assessments:

Open vs. closed, web-based vs. face-to-face assessments: Mention how participants were recruited (online vs. offline), e.g., from an open access website or from a clinic, and clarify if this was a purely web-based trial, or there were face-to-face components (as part of the intervention or for assessment), i.e., to what degree got the study team to know the participant. In online-only trials, clarify if participants were quasi-anonymous and whether having multiple identities was possible or whether technical or logistical measures (e.g., cookies, email confirmation, phone calls) were used to detect/prevent these.

	1	2	3	4	5	
subitem not at all important	○	○	◉	○	○	essential

Clear selection

Does your paper address subitem 4a-ii? *

Copy and paste relevant sections from the manuscript (include quotes in quotation marks "like this" to indicate direct quotes from your manuscript), or elaborate on this item by providing additional information not in the ms, or briefly explain why the item is not applicable/relevant for your study

"recruited either through email and social media outreach invitations or from waiting lists of campus mental health clinics"

4a-iii) Information giving during recruitment

Information given during recruitment. Specify how participants were briefed for recruitment and in the informed consent procedures (e.g., publish the informed consent documentation as appendix, see also item X26), as this information may have an effect on user self-selection, user expectation and may also bias results.

	1	2	3	4	5	
subitem not at all important	○	○	◉	○	○	essential

Clear selection

Does your paper address subitem 4a-iii?

Copy and paste relevant sections from the manuscript (include quotes in quotation marks "like this" to indicate direct quotes from your manuscript), or elaborate on this item by providing additional information not in the ms, or briefly explain why the item is not applicable/relevant for your study

For students recruited from the university mental health clinics, the intake person at the clinic identified potential study participants when the participants asked for an appointment, described the study and answered any questions. Subsequently, interested students were sent an email invitation and link to the online informed consent and baseline survey. Students recruited from the representative samples generated from the list of enrolled students or from those who responded to announcements on the university's social media platforms received an email that described the stud, contact details to answer questions or receive an explanation from the study team and a link to the online informed consent and baseline survey.

4b) Settings and locations where the data were collected

Does your paper address CONSORT subitem 4b? *

Copy and paste relevant sections from the manuscript (include quotes in quotation marks "like this" to indicate direct quotes from your manuscript), or elaborate on this item by providing additional information not in the ms, or briefly explain why the item is not applicable/relevant for your study

“from seven universities in Colombia and Mexico"

4b-i) Report if outcomes were (self-)assessed through online questionnaires

Clearly report if outcomes were (self-)assessed through online questionnaires (as common in web-based trials) or otherwise.

	1	2	3	4	5	
subitem not at all important	○	○	◉	○	○	essential

Clear selection

Does your paper address subitem 4b-i? *

Copy and paste relevant sections from the manuscript (include quotes in quotation marks "like this" to indicate direct quotes from your manuscript), or elaborate on this item by providing additional information not in the ms, or briefly explain why the item is not applicable/relevant for your study

"Two follow-up online self-administered questionnaires (SAQ) were administered 3- and 12-months post-randomization"

4b-ii) Report how institutional affiliations are displayed

Report how institutional affiliations are displayed to potential participants [on ehealth media], as affiliations with prestigious hospitals or universities may affect volunteer rates, use, and reactions with regards to an intervention.(Not a required item – describe only if this may bias results)

	1	2	3	4	5	
subitem not at all important	○	○	○	○	○	essential

Does your paper address subitem 4b-ii?

Copy and paste relevant sections from the manuscript (include quotes in quotation marks "like this" to indicate direct quotes from your manuscript), or elaborate on this item by providing additional information not in the ms, or briefly explain why the item is not applicable/relevant for your study

The institutional logo of the participating university the student belonged to as well as the logo of the National Institute of Psychiatry were displayed, but not considered to have biases results.

5) The interventions for each group with sufficient details to allow replication, including how and when they were actually administered

5-i) Mention names, credential, affiliations of the developers, sponsors, and owners

Mention names, credential, affiliations of the developers, sponsors, and owners [6] (if authors/evaluators are owners or developer of the software, this needs to be declared in a "Conflict of interest" section or mentioned elsewhere in the manuscript).

	1	2	3	4	5	
subitem not at all important	○	○	○	○	○	essential

Does your paper address subitem 5-i?

Copy and paste relevant sections from the manuscript (include quotes in quotation marks "like this" to indicate direct quotes from your manuscript), or elaborate on this item by providing additional information not in the ms, or briefly explain why the item is not applicable/relevant for your study

We refer to the developers of the intervention, and the sponsors, yet there is no conflict of interest to declare.

5-ii) Describe the history/development process

Describe the history/development process of the application and previous formative evaluations (e.g., focus groups, usability testing), as these will have an impact on adoption/use rates and help with interpreting results.

	1	2	3	4	5	
subitem not at all important	○	◉	○	○	○	essential

Clear selection

Does your paper address subitem 5-ii?

Copy and paste relevant sections from the manuscript (include quotes in quotation marks "like this" to indicate direct quotes from your manuscript), or elaborate on this item by providing additional information not in the ms, or briefly explain why the item is not applicable/relevant for your study

We did not develop this intervention, it was developed by SilverCloud Health. We did a cultural adaptation using focus groups.

5-iii) Revisions and updating

Revisions and updating. Clearly mention the date and/or version number of the application/intervention (and comparator, if applicable) evaluated, or describe whether the intervention underwent major changes during the evaluation process, or whether the development and/or content was "frozen" during the trial. Describe dynamic components such as news feeds or changing content which may have an impact on the replicability of the intervention (for unexpected events see item 3b).

	1	2	3	4	5	
subitem not at all important	○	◉	○	○	○	essential

Clear selection

Does your paper address subitem 5-iii?

Copy and paste relevant sections from the manuscript (include quotes in quotation marks "like this" to indicate direct quotes from your manuscript), or elaborate on this item by providing additional information not in the ms, or briefly explain why the item is not applicable/relevant for your study

We did a cultural adaptation prior to implementing the trial (which is the focus of a manuscript currently under review), no revisions or updates were made during the trial.

5-iv) Quality assurance methods

Provide information on quality assurance methods to ensure accuracy and quality of information provided [1], if applicable.

	1	2	3	4	5	
subitem not at all important	○	○	○	○	○	essential

Does your paper address subitem 5-iv?

Copy and paste relevant sections from the manuscript (include quotes in quotation marks "like this" to indicate direct quotes from your manuscript), or elaborate on this item by providing additional information not in the ms, or briefly explain why the item is not applicable/relevant for your study

Quality assurance methods were not employed as both the self-administered questionnaire and the intervention were highly structured and standardized. Guidance was supervised weekly by a trained clinical (PhD level) psychologist.

5-v) Ensure replicability by publishing the source code, and/or providing screenshots/screen-capture video, and/or providing flowcharts of the algorithms used

Ensure replicability by publishing the source code, and/or providing screenshots/screen-capture video, and/or providing flowcharts of the algorithms used. Replicability (i.e., other researchers should in principle be able to replicate the study) is a hallmark of scientific reporting.

	1	2	3	4	5	
subitem not at all important	○	○	◉	○	○	essential

Clear selection

Does your paper address subitem 5-v?

Copy and paste relevant sections from the manuscript (include quotes in quotation marks "like this" to indicate direct quotes from your manuscript), or elaborate on this item by providing additional information not in the ms, or briefly explain why the item is not applicable/relevant for your study

We did not provide source codes for data analysis, visual documentation of the intervention, or algorithm flowcharts. However, source codes for data analysis are available upon reasonable request.

5-vi) Digital preservation

Digital preservation: Provide the URL of the application, but as the intervention is likely to change or disappear over the course of the years; also make sure the intervention is archived (Internet Archive, webcitation.org, and/or publishing the source code or screenshots/videos alongside the article). As pages behind login screens cannot be archived, consider creating demo pages which are accessible without login.

	1	2	3	4	5	
subitem not at all important	○	○	○	○	○	essential

Does your paper address subitem 5-vi?

Copy and paste relevant sections from the manuscript (include quotes in quotation marks "like this" to indicate direct quotes from your manuscript), or elaborate on this item by providing additional information not in the ms, or briefly explain why the item is not applicable/relevant for your study

Because we licensed the intervention, but we are not the owners, we can`t provide this.

5-vii) Access

Access: Describe how participants accessed the application, in what setting/context, if they had to pay (or were paid) or not, whether they had to be a member of specific group. If known, describe how participants obtained “access to the platform and Internet” [1]. To ensure access for editors/reviewers/readers, consider to provide a “backdoor” login account or demo mode for reviewers/readers to explore the application (also important for archiving purposes, see vi).

	1	2	3	4	5	
subitem not at all important	○	○	◉	○	○	essential

Clear selection

Does your paper address subitem 5-vii? *

Copy and paste relevant sections from the manuscript (include quotes in quotation marks "like this" to indicate direct quotes from your manuscript), or elaborate on this item by providing additional information not in the ms, or briefly explain why the item is not applicable/relevant for your study

Participants accessed the application free of charge via a personalized link if they were a student from one of the participating universities and met inclusion criteria.

5-viii) Mode of delivery, features/functionalities/components of the intervention and comparator, and the theoretical framework

Describe mode of delivery, features/functionalities/components of the intervention and comparator, and the theoretical framework [6] used to design them (instructional strategy [1], behaviour change techniques, persuasive features, etc., see e.g., [7, 8] for terminology). This includes an in-depth description of the content (including where it is coming from and who developed it) [1],” whether [and how] it is tailored to individual circumstances and allows users to track their progress and receive feedback” [6]. This also includes a description of communication delivery channels and – if computer-mediated communication is a component – whether communication was synchronous or asynchronous [6]. It also includes information on presentation strategies [1], including page design principles, average amount of text on pages, presence of hyperlinks to other resources, etc. [1].

	1	2	3	4	5	
subitem not at all important	○	○	○	○	◉	essential

Clear selection

Does your paper address subitem 5-viii? *

Copy and paste relevant sections from the manuscript (include quotes in quotation marks "like this" to indicate direct quotes from your manuscript), or elaborate on this item by providing additional information not in the ms, or briefly explain why the item is not applicable/relevant for your study

“The wb-CBT program (both guided and self-guided modalities) was a culturally adapted version of SilverCloud Health by Amwell’s Space from Anxiety and Depression program, a transdiagnostic wb-CBT program that can be accessed by computer, tablet, or mobile phone and that has been found to be effective in treating anxiety and depression [21-23]. The content is identical in the guided and self-guided programs, but participants assigned to the guided program receive during the first 8 weeks after randomization weekly asynchronous written messages through the platform from trained BA-level coaches with a degree in psychology intended to generate personalized experiences and offer feedback [24]. The intervention has seven primary modules that focus on cognitive restructuring, behavioral activation and relaxation techniques and several other ancillary modules (e.g., sleep, anger). The program content, which includes videos, audios, exercises, and vignettes, is intended for completion within 8 weeks, although participants have continued access to the program for initial use or refresher reviews for 12 months after randomization (described in more detail in [16]). TAU consisted of referral to the clinic in the three universities that had student mental health clinics and referral to informal counseling services in the other universities that faculty provide to place students having anxiety or depression with community treatment providers. As randomization occurred during the COVID-19 pandemic lockdown, most university services were offered solely through videoconferencing platforms during the study.”

5-ix) Describe use parameters

Describe use parameters (e.g., intended “doses” and optimal timing for use). Clarify what instructions or recommendations were given to the user, e.g., regarding timing, frequency, heaviness of use, if any, or was the intervention used ad libitum.

	1	2	3	4	5	
subitem not at all important	○	○	○	○	○	essential

Does your paper address subitem 5-ix?

Copy and paste relevant sections from the manuscript (include quotes in quotation marks "like this" to indicate direct quotes from your manuscript), or elaborate on this item by providing additional information not in the ms, or briefly explain why the item is not applicable/relevant for your study

Participants were told to complete the program at their own pace, but it was recommended to complete one module per week.

5-x) Clarify the level of human involvement

Clarify the level of human involvement (care providers or health professionals, also technical assistance) in the e-intervention or as co-intervention (detail number and expertise of professionals involved, if any, as well as “type of assistance offered, the timing and frequency of the support, how it is initiated, and the medium by which the assistance is delivered”. It may be necessary to distinguish between the level of human involvement required for the trial, and the level of human involvement required for a routine application outside of a RCT setting (discuss under item 21 – generalizability).

	1	2	3	4	5	
subitem not at all important	○	○	○	○	○	essential

Does your paper address subitem 5-x?

Copy and paste relevant sections from the manuscript (include quotes in quotation marks "like this" to indicate direct quotes from your manuscript), or elaborate on this item by providing additional information not in the ms, or briefly explain why the item is not applicable/relevant for your study

“Participants assigned to the guided program receive during the first 8 weeks after randomization weekly asynchronous written messages through the platform from trained BA-level coaches with a degree in psychology intended to generate personalized experiences and offer feedback.”

5-xi) Report any prompts/reminders used

Report any prompts/reminders used: Clarify if there were prompts (letters, emails, phone calls, SMS) to use the application, what triggered them, frequency etc. It may be necessary to distinguish between the level of prompts/reminders required for the trial, and the level of prompts/reminders for a routine application outside of a RCT setting (discuss under item 21 – generalizability).

	1	2	3	4	5	
subitem not at all important	○	◉	○	○	○	essential

Clear selection

Does your paper address subitem 5-xi? *

Copy and paste relevant sections from the manuscript (include quotes in quotation marks "like this" to indicate direct quotes from your manuscript), or elaborate on this item by providing additional information not in the ms, or briefly explain why the item is not applicable/relevant for your study

Participants could activate reminders in the app if they chose to do so and choose the days/ times of the reminders and the mode of reminders (email or a message in the platform).

5-xii) Describe any co-interventions (incl. training/support)

Describe any co-interventions (incl. training/support): Clearly state any interventions that are provided in addition to the targeted eHealth intervention, as ehealth intervention may not be designed as stand-alone intervention. This includes training sessions and support [1]. It may be necessary to distinguish between the level of training required for the trial, and the level of training for a routine application outside of a RCT setting (discuss under item 21 – generalizability.

	1	2	3	4	5	
subitem not at all important	○	○	○	○	○	essential

Does your paper address subitem 5-xii? *

Copy and paste relevant sections from the manuscript (include quotes in quotation marks "like this" to indicate direct quotes from your manuscript), or elaborate on this item by providing additional information not in the ms, or briefly explain why the item is not applicable/relevant for your study

There were no additional co-interventions.

6a) Completely defined pre-specified primary and secondary outcome measures, including how and when they were assessed

Does your paper address CONSORT subitem 6a? *

Copy and paste relevant sections from the manuscript (include quotes in quotation marks "like this" to indicate direct quotes from your manuscript), or elaborate on this item by providing additional information not in the ms, or briefly explain why the item is not applicable/relevant for your study

“Anxiety was measured with the self-report GAD-7 [19] and depression with the self-report PHQ-9 [20], both of which are commonly used in psychotherapy trials and have good psychometric properties [25] and have been shown previously to have strong convergent validity with other anxiety and depression scales in Colombia [26, 27] and Mexico [28, 29]. These scales are often combined into a single scale to measure anxiety and depression in the Patient Health Questionnaire Anxiety and Depression Scale (PHQ-ADS; [25]). We examined joint remission (the conjunction of GAD-7=0-4 and PHQ-9=0-4) as well as mean PHQ-ADS scores at 3 and 12 months. “

6a-i) Online questionnaires: describe if they were validated for online use and apply CHERRIES items to describe how the questionnaires were designed/deployed

If outcomes were obtained through online questionnaires, describe if they were validated for online use and apply CHERRIES items to describe how the questionnaires were designed/deployed [9].

	1	2	3	4	5	
subitem not at all important	○	○	●	○	○	essential

Clear selection

Does your paper address subitem 6a-i?

Copy and paste relevant sections from manuscript text

“Anxiety was measured with the self-report GAD-7 [19] and depression with the self-report PHQ-9 [20], both of which are commonly used in psychotherapy trials and have good psychometric properties [25] and have been shown previously to have strong convergent validity with other anxiety and depression scales in Colombia [26, 27] and Mexico [28, 29].”

6a-ii) Describe whether and how “use” (including intensity of use/dosage) was defined/measured/monitored

Describe whether and how “use” (including intensity of use/dosage) was defined/measured/monitored (logins, logfile analysis, etc.). Use/adoption metrics are important process outcomes that should be reported in any ehealth trial.

	1	2	3	4	5	
subitem not at all important	○	○	○	○	●	essential

Clear selection

Does your paper address subitem 6a-ii?

Copy and paste relevant sections from manuscript text

“Metadata from the online SilverCloud Health intervention portal provided usage metrics to measure compliance. Such compliance metrics are often used in analyses of web-based intervention trials [30, 31]. We focused on the number of minutes each participant spent logged on each week after the end of the guidance phase of the intervention over the course of 12 months as our primary measure of compliance, as adequate time has been a consistent predictor of higher adherence to wb-CBTs and other web-based mental health interventions [32]. “

6a-iii) Describe whether, how, and when qualitative feedback from participants was obtained

Describe whether, how, and when qualitative feedback from participants was obtained (e.g., through emails, feedback forms, interviews, focus groups).

	1	2	3	4	5	
subitem not at all important	○	○	◉	○	○	essential

Clear selection

Does your paper address subitem 6a-iii?

Copy and paste relevant sections from manuscript text

Participants could provide feedback at the end of each program module in the platform. This qualitative data was not included in this manuscript.

6b) Any changes to trial outcomes after the trial commenced, with reasons

Does your paper address CONSORT subitem 6b? *

Copy and paste relevant sections from the manuscript (include quotes in quotation marks "like this" to indicate direct quotes from your manuscript), or elaborate on this item by providing additional information not in the ms, or briefly explain why the item is not applicable/relevant for your study

No changes were made to trial outcomes.

7a) How sample size was determined

NPT: When applicable, details of whether and how the clustering by care provides or centers was addressed

7a-i) Describe whether and how expected attrition was taken into account when calculating the sample size

Describe whether and how expected attrition was taken into account when calculating the sample size.

	1	2	3	4	5	
subitem not at all important	○	○	○	○	○	essential

Does your paper address subitem 7a-i?

Copy and paste relevant sections from manuscript title (include quotes in quotation marks "like this" to indicate direct quotes from your manuscript), or elaborate on this item by providing additional information not in the ms, or briefly explain why the item is not applicable/relevant for your study

We powered the study to estimate heterogeneity of treatment effects (HTE) as defined by the increase in the proportion of patients we would expect to remit from the primary outcomes based on optimized rather than randomized treatment of 5%. We carried out such a simulation by building a series of hypothetical population models that assumed that each patient was randomized either to i-CBT or a control condition; that the aggregate remission rates after adjusting for loss to follow-up were 27.5% and 17.5% in the two treatment arms, respectively; and that a series of relatively complex nonlinear-interactive multivariate associations existed between 20 predictors and remission. The assumed distributions of predictors and interaction values among predictors were consistent with those found in previous studies. We varied these coefficient values in different models to maintain the same aggregate remission rates but to have the difference between remission rates based on randomized versus optimized treatment vary between 0 and 15%. In each model, the remission rate if intervention assignment was randomized was set at 10% (i.e., 27.5–17.5%), but the remission rate if intervention assignment was optimized (i.e., if each student who would have a better outcome if assigned to i-CBT was, in fact, assigned to i-CBT and all other students were assigned to the control condition, which in some cases could involve these students receiving treatment at the student clinic and having better outcomes than if they received i-CBT) was varied between 0 and 15%. We discovered that adequate statistical power (again defined as .8 power based on .05-level two-sided tests) to detect a difference between the two remission rates of 5%, which we considered the minimum difference of interest, would require a sample of 500 patients per treatment arm. A difference of 5% would represent a 50% increase on the 10% aggregate base of the difference in the remission rate between intervention and control groups.

7b) When applicable, explanation of any interim analyses and stopping guidelines

Does your paper address CONSORT subitem 7b? *

Copy and paste relevant sections from the manuscript (include quotes in quotation marks "like this" to indicate direct quotes from your manuscript), or elaborate on this item by providing additional information not in the ms, or briefly explain why the item is not applicable/relevant for your study

Interim analyses assessed adequate randomization. There were no stopping guidelines.

8a) Method used to generate the random allocation sequence

NPT: When applicable, how care providers were allocated to each trial group

Does your paper address CONSORT subitem 8a? *

Copy and paste relevant sections from the manuscript (include quotes in quotation marks "like this" to indicate direct quotes from your manuscript), or elaborate on this item by providing additional information not in the ms, or briefly explain why the item is not applicable/relevant for your study

Randomization was generated by the Qualitrics software package.

8b) Type of randomisation; details of any restriction (such as blocking and block size)

Does your paper address CONSORT subitem 8b? *

Copy and paste relevant sections from the manuscript (include quotes in quotation marks "like this" to indicate direct quotes from your manuscript), or elaborate on this item by providing additional information not in the ms, or briefly explain why the item is not applicable/relevant for your study

Participants were block randomized, with stratification by sex and severity of baseline anxiety and depression and equal allocation across the 3 intervention arms.

9) Mechanism used to implement the random allocation sequence (such as sequentially numbered containers), describing any steps taken to conceal the sequence until interventions were assigned

Does your paper address CONSORT subitem 9? *

Copy and paste relevant sections from the manuscript (include quotes in quotation marks "like this" to indicate direct quotes from your manuscript), or elaborate on this item by providing additional information not in the ms, or briefly explain why the item is not applicable/relevant for your study

Randomization was performed by the Qualtrics software in which the participants responded to the baseline questionnaire. The allocation sequence was automatically generated in the online baseline self-administered questionnaire platform (Qualitrics) (and thus concealed) stratifying by the three-way cross-classification of sex at birth, anxiety symptom severity, and depression symptom severity.

10) Who generated the random allocation sequence, who enrolled participants, and who assigned participants to interventions

Does your paper address CONSORT subitem 10? *

Copy and paste relevant sections from the manuscript (include quotes in quotation marks "like this" to indicate direct quotes from your manuscript), or elaborate on this item by providing additional information not in the ms, or briefly explain why the item is not applicable/relevant for your study

Randomization was performed by the Qualtrics software in which the participants responded to the baseline questionnaire. Once the software assigned the participants a screen was shown to the participant which explained which intervention he/she was assigned to and how to proceed. This was all automated without human involvement.

11a) If done, who was blinded after assignment to interventions (for example, participants, care providers, those assessing outcomes) and how

NPT: Whether or not administering co-interventions were blinded to group assignment

11a-i) Specify who was blinded, and who wasn't

Specify who was blinded, and who wasn't. Usually, in web-based trials it is not possible to blind the participants [1, 3] (this should be clearly acknowledged), but it may be possible to blind outcome assessors, those doing data analysis or those administering co-interventions (if any).

	1	2	3	4	5	
subitem not at all important	○	○	◉	○	○	essential

Clear selection

Does your paper address subitem 11a-i? *

Copy and paste relevant sections from the manuscript (include quotes in quotation marks "like this" to indicate direct quotes from your manuscript), or elaborate on this item by providing additional information not in the ms, or briefly explain why the item is not applicable/relevant for your study

Participants were not blinded to treatment arm; the data analyst was blinded.

11a-ii) Discuss e.g., whether participants knew which intervention was the "intervention of interest" and which one was the "comparator"

Informed consent procedures (4a-ii) can create biases and certain expectations - discuss e.g., whether participants knew which intervention was the "intervention of interest" and which one was the "comparator".

	1	2	3	4	5	
subitem not at all important	○	○	◉	○	○	essential

Clear selection

Does your paper address subitem 11a-ii?

Copy and paste relevant sections from the manuscript (include quotes in quotation marks "like this" to indicate direct quotes from your manuscript), or elaborate on this item by providing additional information not in the ms, or briefly explain why the item is not applicable/relevant for your study

Participants were told that different interventions work better for different people and thus there was no detectable intervention of interest.

11b) If relevant, description of the similarity of interventions

(this item is usually not relevant for ehealth trials as it refers to similarity of a placebo or sham intervention to a active medication/intervention)

Does your paper address CONSORT subitem 11b? *

Copy and paste relevant sections from the manuscript (include quotes in quotation marks "like this" to indicate direct quotes from your manuscript), or elaborate on this item by providing additional information not in the ms, or briefly explain why the item is not applicable/relevant for your study

“The content is identical in the guided and self-guided programs, but participants assigned to the guided program receive during the first 8 weeks after randomization weekly asynchronous written messages through the platform from trained BA-level coaches with a degree in psychology intended to generate personalized experiences and offer feedback [24].”

12a) Statistical methods used to compare groups for primary and secondary outcomes

NPT: When applicable, details of whether and how the clustering by care providers or centers was addressed

Does your paper address CONSORT subitem 12a? *

Copy and paste relevant sections from the manuscript (include quotes in quotation marks "like this" to indicate direct quotes from your manuscript), or elaborate on this item by providing additional information not in the ms, or briefly explain why the item is not applicable/relevant for your study

“Our primary outcome, joint anxiety-depression remission, was defined as follow-up scores of 0-4 on both the GAD-7 and PHQ-9. Analysis estimated these joint remission rates by adjusting for nonrandom loss to follow-up and then calculated adjusted risk differences (ARD) across arms. In our analysis of mean symptom changes, in comparison, we estimated baseline, 3-month, and 12-month means, again adjusting for loss to follow-up, and then calculated adjusted mean differences (AMD) across arms. “

12a-i) Imputation techniques to deal with attrition / missing values

Imputation techniques to deal with attrition / missing values: Not all participants will use the intervention/comparator as intended and attrition is typically high in ehealth trials. Specify how participants who did not use the application or dropped out from the trial were treated in the statistical analysis (a complete case analysis is strongly discouraged, and simple imputation techniques such as LOCF may also be problematic [4]).

	1	2	3	4	5	
subitem not at all important	○	○	○	○	◉	essential

Clear selection

Does your paper address subitem 12a-i? *

Copy and paste relevant sections from the manuscript (include quotes in quotation marks "like this" to indicate direct quotes from your manuscript), or elaborate on this item by providing additional information not in the ms, or briefly explain why the item is not applicable/relevant for your study

“All ATE analyses incorporated adjustments for loss to follow-up using a doubly robust estimation method that combined outcome modeling (similar to imputing missing values) and propensity score modeling through the longitudinal Targeted Minimum Loss-based Estimation (TMLE) method [33]. For the 3-month analysis, we employed the tmle3 package, whereas for the 12-month analysis, we utilized ltmle [34, 35]. The latter program allows information about partial follow-up (in our case, response to the 3-month follow-up but not to the 12-month follow-up) to be used in adjusting for loss to follow-up. We used the mice R package [36, 37] for multiple imputation with predictive mean matching to predict 3-month follow-up results for participants without 3-month data but possessing 12-month data.”

12b) Methods for additional analyses, such as subgroup analyses and adjusted analyses

Does your paper address CONSORT subitem 12b? *

Copy and paste relevant sections from the manuscript (include quotes in quotation marks "like this" to indicate direct quotes from your manuscript), or elaborate on this item by providing additional information not in the ms, or briefly explain why the item is not applicable/relevant for your study

“We noted in the introduction that the stronger effects typically found for guided than self-guided wb-CBT are typically interpreted as due to the higher compliance of participants randomized to guided than self-guided wb-CBT. However, there is often no test of this assumption in wb-CBT trials… given our interest in heterogeneity, we used the rich set of baseline covariates in our trial to develop a machine learning model that predicted observed compliance among participants randomized to self-guided wb-CBT. We did this using the Super Learner (SL) R program [42] to train a nested 10-fold cross-validated (10F-CV) ensemble machine learning model. Given that the baseline assessment was obtained for all participants across all three arms, individual-level predicted compliance scores could then be generated for all participants in the trial regardless of the arm to which they were randomized. This allowed us to define predicted compliance based on information available prior to randomization and to make sure individual-level scores in the self-guided arm were not biased by knowledge of the participant’s observed compliance by virtue of the use of nested 10F-CV. Predicted compliance was then used as a specifier in evaluating comparative intervention effects in subgroups defined by the likelihood that participants would continue to comply with self-guided wb-CBT over the full 12-month follow-up period if they were assigned to it. This approach has been recommended as the best way to study baseline predictors of heterogeneity based on differential compliance in cases where heterogeneity is found [43]. The kernel Shapley Additive Explanations (SHAP) method [41], applied through the fastshap R package [42], was then used to examine which baseline predictors played the largest role in defining predicted compliance. SHAP values are created by calculating one at a time for each significant predictor the extent to which predicted outcome scores change under the prediction model when the score on the predictor is changed from its observed value to the mean value in the sample. The SHAP value for a given predictor is defined as the mean of the absolute value of this difference across all participants. We report the proportional SHAP value (SHAPP), the SHAP value for the individual predictor divided by the SHAP value for the entire model (i.e., the effect of setting all predictors to their mean values for all participants). SHAPP values for individual predictors can sum to more than 100% because most people have values on some predictors above the mean and others below the mean. “

X26) REB/IRB Approval and Ethical Considerations [recommended as subheading under "Methods"] (not a CONSORT item)

X26-i) Comment on ethics committee approval

	1	2	3	4	5	
subitem not at all important	○	○	○	○	◉	essential

Clear selection

Does your paper address subitem X26-i?

Copy and paste relevant sections from the manuscript (include quotes in quotation marks "like this" to indicate direct quotes from your manuscript), or elaborate on this item by providing additional information not in the ms, or briefly explain why the item is not applicable/relevant for your study

"This study protocol was reviewed and approved by the Institutional Review Boards of the Instituto Nacional de Psiquiatria (National Institute of Psychiatry) Ramón de la Fuente-Muñiz in Mexico, approval number [CEI/C/015/2020] and Harvard Medical School in the United States, approval number [20-1494]."

x26-ii) Outline informed consent procedures

Outline informed consent procedures e.g., if consent was obtained offline or online (how? Checkbox, etc.?), and what information was provided (see 4a-ii). See [6] for some items to be included in informed consent documents.

	1	2	3	4	5	
subitem not at all important	○	○	○	○	◉	essential

Clear selection

Does your paper address subitem X26-ii?

Copy and paste relevant sections from the manuscript (include quotes in quotation marks "like this" to indicate direct quotes from your manuscript), or elaborate on this item by providing additional information not in the ms, or briefly explain why the item is not applicable/relevant for your study

“Online informed consent was obtained from participants.”

X26-iii) Safety and security procedures

Safety and security procedures, incl. privacy considerations, and any steps taken to reduce the likelihood or detection of harm (e.g., education and training, availability of a hotline)

	1	2	3	4	5	
subitem not at all important	○	○	◉	○	○	essential

Clear selection

Does your paper address subitem X26-iii?

Copy and paste relevant sections from the manuscript (include quotes in quotation marks "like this" to indicate direct quotes from your manuscript), or elaborate on this item by providing additional information not in the ms, or briefly explain why the item is not applicable/relevant for your study

“Exclusion criteria were reporting recent suicidal ideation with intent or screening positive for a history of bipolar disorder or nonaffective psychosis. Students who were excluded from the trial were contacted by a clinical liaison at their university and provided appropriate referrals.”

RESULTS

13a) For each group, the numbers of participants who were randomly assigned, received intended treatment, and were analysed for the primary outcome

NPT: The number of care providers or centers performing the intervention in each group and the number of patients treated by each care provider in each center

Does your paper address CONSORT subitem 13a? *

Copy and paste relevant sections from the manuscript (include quotes in quotation marks "like this" to indicate direct quotes from your manuscript), or elaborate on this item by providing additional information not in the ms, or briefly explain why the item is not applicable/relevant for your study

"Figure 1 shows the CONSORT diagram of the enrollment, allocation, 3-month and 12-month follow-up rates of trial participants."

13b) For each group, losses and exclusions after randomisation, together with reasons

Does your paper address CONSORT subitem 13b? (NOTE: Preferably, this is shown in a CONSORT flow diagram) *

Copy and paste relevant sections from the manuscript (include quotes in quotation marks "like this" to indicate direct quotes from your manuscript), or elaborate on this item by providing additional information not in the ms, or briefly explain why the item is not applicable/relevant for your study

Refer to Figure 1.

13b-i) Attrition diagram

Strongly recommended: An attrition diagram (e.g., proportion of participants still logging in or using the intervention/comparator in each group plotted over time, similar to a survival curve) or other figures or tables demonstrating usage/dose/engagement.

	1	2	3	4	5	
subitem not at all important	○	○	○	○	◉	essential

Clear selection

Does your paper address subitem 13b-i?

Copy and paste relevant sections from the manuscript or cite the figure number if applicable (include quotes in quotation marks "like this" to indicate direct quotes from your manuscript), or elaborate on this item by providing additional information not in the ms, or briefly explain why the item is not applicable/relevant for your study

Refer to Table S4 in Multimedia Appendix 1.

14a) Dates defining the periods of recruitment and follow-up

Does your paper address CONSORT subitem 14a? *

Copy and paste relevant sections from the manuscript (include quotes in quotation marks "like this" to indicate direct quotes from your manuscript), or elaborate on this item by providing additional information not in the ms, or briefly explain why the item is not applicable/relevant for your study

Study enrollment took place between March 1 and October 26, 2021.

14a-i) Indicate if critical “secular events” fell into the study period

Indicate if critical “secular events” fell into the study period, e.g., significant changes in Internet resources available or “changes in computer hardware or Internet delivery resources”

	1	2	3	4	5	
subitem not at all important	(selected)	○	○	○	○	essential

Clear selection

Does your paper address subitem 14a-i?

Copy and paste relevant sections from the manuscript (include quotes in quotation marks "like this" to indicate direct quotes from your manuscript), or elaborate on this item by providing additional information not in the ms, or briefly explain why the item is not applicable/relevant for your study

Not applicable.

14b) Why the trial ended or was stopped (early)

Does your paper address CONSORT subitem 14b? *

Copy and paste relevant sections from the manuscript (include quotes in quotation marks "like this" to indicate direct quotes from your manuscript), or elaborate on this item by providing additional information not in the ms, or briefly explain why the item is not applicable/relevant for your study

Not applicable.

15) A table showing baseline demographic and clinical characteristics for each group

NPT: When applicable, a description of care providers (case volume, qualification, expertise, etc.) and centers (volume) in each group

Does your paper address CONSORT subitem 15? *

Copy and paste relevant sections from the manuscript (include quotes in quotation marks "like this" to indicate direct quotes from your manuscript), or elaborate on this item by providing additional information not in the ms, or briefly explain why the item is not applicable/relevant for your study

Refer to Table 1.

15-i) Report demographics associated with digital divide issues

In ehealth trials it is particularly important to report demographics associated with digital divide issues, such as age, education, gender, social-economic status, computer/Internet/ehealth literacy of the participants, if known.

	1	2	3	4	5	
subitem not at all important	○	○	◉	○	○	essential

Clear selection

Does your paper address subitem 15-i? *

Copy and paste relevant sections from the manuscript (include quotes in quotation marks "like this" to indicate direct quotes from your manuscript), or elaborate on this item by providing additional information not in the ms, or briefly explain why the item is not applicable/relevant for your study

Refer to Table 1.

16) For each group, number of participants (denominator) included in each analysis and whether the analysis was by original assigned groups

16-i) Report multiple “denominators” and provide definitions

Report multiple “denominators” and provide definitions: Report N’s (and effect sizes) “across a range of study participation [and use] thresholds” [1], e.g., N exposed, N consented, N used more than x times, N used more than y weeks, N participants “used” the intervention/comparator at specific pre-defined time points of interest (in absolute and relative numbers per group). Always clearly define “use” of the intervention.

	1	2	3	4	5	
subitem not at all important	○	○	○	○	◉	essential

Clear selection

Does your paper address subitem 16-i? *

Copy and paste relevant sections from the manuscript (include quotes in quotation marks "like this" to indicate direct quotes from your manuscript), or elaborate on this item by providing additional information not in the ms, or briefly explain why the item is not applicable/relevant for your study

Refer to all tables.

16-ii) Primary analysis should be intent-to-treat

Primary analysis should be intent-to-treat, secondary analyses could include comparing only “users”, with the appropriate caveats that this is no longer a randomized sample (see 18-i).

	1	2	3	4	5	
subitem not at all important	○	○	◉	○	○	essential

Clear selection

Does your paper address subitem 16-ii?

Copy and paste relevant sections from the manuscript (include quotes in quotation marks "like this" to indicate direct quotes from your manuscript), or elaborate on this item by providing additional information not in the ms, or briefly explain why the item is not applicable/relevant for your study

Refer to the “Data Analysis” section.

17a) For each primary and secondary outcome, results for each group, and the estimated effect size and its precision (such as 95% confidence interval)

Does your paper address CONSORT subitem 17a? *

Copy and paste relevant sections from the manuscript (include quotes in quotation marks "like this" to indicate direct quotes from your manuscript), or elaborate on this item by providing additional information not in the ms, or briefly explain why the item is not applicable/relevant for your study

"Joint anxiety-depression remission rates differed significantly across intervention arms at both 3 months (37.2%–50.3%; χ22 = 12.4, P=.002) and 12 months (32.5%–38.9%; χ22 = 7.6, P=.02) (Table 2 Part I). As we reported in a previous publication [16], at 3 months the remission rate of guided wb-CBT was significantly higher than that of either self-guided wb-CBT (ARD = 13.1%, χ21 = 10.4, P=.001) or TAU (ARD = 11.2%, χ21 = 8.4, P=.004), but the remission rates were non-significantly different between self-guided wb-CBT and TAU (ARD = -1.9%, χ22 = 0.2, P=.63). The new results for 12 months reported here for the first time, in comparison, show that the remission rate of self-guided wb-CBT was significantly higher than that of either guided wb-CBT (ARD = -6.0%, χ21 = 4.9, P=.03) or TAU (ARD = -6.5%, χ21 = 6.3, P=.01), whereas the remission rates were non-significantly different between guided wb-CBT and TAU (ARD = 0.4%, χ21 = 0.03, P=.86)."

17a-i) Presentation of process outcomes such as metrics of use and intensity of use

In addition to primary/secondary (clinical) outcomes, the presentation of process outcomes such as metrics of use and intensity of use (dose, exposure) and their operational definitions is critical. This does not only refer to metrics of attrition (13-b) (often a binary variable), but also to more continuous exposure metrics such as "average session length". These must be accompanied by a technical description how a metric like a "session" is defined (e.g., timeout after idle time) [1] (report under item 6a).

	1	2	3	4	5	
subitem not at all important	○	○	○	○	◉	essential

Clear selection

Does your paper address subitem 17a-i?

Copy and paste relevant sections from the manuscript (include quotes in quotation marks "like this" to indicate direct quotes from your manuscript), or elaborate on this item by providing additional information not in the ms, or briefly explain why the item is not applicable/relevant for your study

“Metadata from the online SilverCloud Health intervention portal provided usage metrics to measure compliance. Such compliance metrics are often used in analyses of web-based intervention trials [30, 31]. We focused on the number of minutes each participant spent logged on each week after the end of the guidance phase of the intervention over the course of 12 months as our primary measure of compliance, as adequate time has been a consistent predictor of higher adherence to wb-CBTs and other web-based mental health interventions [32].”

17b) For binary outcomes, presentation of both absolute and relative effect sizes is recommended

Does your paper address CONSORT subitem 17b? *

Copy and paste relevant sections from the manuscript (include quotes in quotation marks "like this" to indicate direct quotes from your manuscript), or elaborate on this item by providing additional information not in the ms, or briefly explain why the item is not applicable/relevant for your study

Refer to Table 3.

18) Results of any other analyses performed, including subgroup analyses and adjusted analyses, distinguishing pre-specified from exploratory

Does your paper address CONSORT subitem 18? *

Copy and paste relevant sections from the manuscript (include quotes in quotation marks "like this" to indicate direct quotes from your manuscript), or elaborate on this item by providing additional information not in the ms, or briefly explain why the item is not applicable/relevant for your study

"Exploratory analyses suggested that the best dichotomous distinction was between the 40% of participants with highest predicted compliance and the remaining 60% of participants. A significant interaction was found between this dichotomy and randomization to self-guided wb-CBT in predicting 12-month joint remission ([illegible]21 = 13.5, P < .001). In the 40% of participants with high predicted compliance, 12-month joint anxiety-depression remission rates varied significantly across arms (χ22 = 17.5, P < .001) due to the remission rate being significantly higher with self-guided wb-CBT than either guided wb-CBT (ARD = -9.3%, χ21 = 7.3, P=.007) or TAU (ARD = 11.0%, χ21 = 19.3, P < .001), whereas there was no significant difference between guided wb-CBT and TAU (ARD = 1.7%, χ21 = 0.2, P=.62). In the 60% of participants with low predicted compliance, in comparison, 12-month joint remission rates did not differ significantly across intervention arms (χ22 = 1.2, P=.54).A similar result was found for 12-month mean PHQ-ADS scores (Table 3), where scores varied significantly across intervention arms in the 40% of participants with high predicted compliance (χ22 = 47.0, P < .001) due to a significantly lower mean for self-guided wb-CBT than either guided wb-CBT (AMD = 4.2, χ21 = 26.2, P < .001) or TAU (AMD = -3.2, χ21 = 30.1, P < .001), but with no significant difference between guided wb-CBT and TAU (AMD = 1.0, χ21 = 1.6, P=.20; Table 3, Part II). In the 60% of participants with low predicted compliance, in comparison, the mean PHQ-ADS score was significantly lower with self-guided wb-CBT than TAU (AMD = -1.8, χ21 = 5.9, P=.02), but mean PHQ-ADS scores did not differ significantly either between self-guided and guided wb-CBT (AMD = 1.0, χ21 = 1.2, P=.28) or between guided wb-CBT and TAU (AMD = -0.8, χ21 = 0.6, P=.43)."

18-i) Subgroup analysis of comparing only users

A subgroup analysis of comparing only users is not uncommon in ehealth trials, but if done, it must be stressed that this is a self-selected sample and no longer an unbiased sample from a randomized trial (see 16-iii).

	1	2	3	4	5	
subitem not at all important	○	○	○	○	◉	essential

Clear selection

Does your paper address subitem 18-i?

Copy and paste relevant sections from the manuscript (include quotes in quotation marks "like this" to indicate direct quotes from your manuscript), or elaborate on this item by providing additional information not in the ms, or briefly explain why the item is not applicable/relevant for your study

Refer to Tables 2 and 3.

19) All important harms or unintended effects in each group
(for specific guidance see CONSORT for harms)

Does your paper address CONSORT subitem 19? *

Copy and paste relevant sections from the manuscript (include quotes in quotation marks "like this" to indicate direct quotes from your manuscript), or elaborate on this item by providing additional information not in the ms, or briefly explain why the item is not applicable/relevant for your study

Not applicable.

19-i) Include privacy breaches, technical problems

Include privacy breaches, technical problems. This does not only include physical “harm” to participants, but also incidents such as perceived or real privacy breaches [1], technical problems, and other unexpected/unintended incidents. “Unintended effects” also includes unintended positive effects [2].

	1	2	3	4	5	
subitem not at all important	○	○	◉	○	○	essential

Clear selection

Does your paper address subitem 19-i?

Copy and paste relevant sections from the manuscript (include quotes in quotation marks "like this" to indicate direct quotes from your manuscript), or elaborate on this item by providing additional information not in the ms, or briefly explain why the item is not applicable/relevant for your study

Not applicable.

19-ii) Include qualitative feedback from participants or observations from staff/researchers

Include qualitative feedback from participants or observations from staff/researchers, if available, on strengths and shortcomings of the application, especially if they point to unintended/unexpected effects or uses. This includes (if available) reasons for why people did or did not use the application as intended by the developers.

	1	2	3	4	5	
subitem not at all important	(•)	( )	( )	( )	( )	essential

Clear selection

Does your paper address subitem 19-ii?

Copy and paste relevant sections from the manuscript (include quotes in quotation marks "like this" to indicate direct quotes from your manuscript), or elaborate on this item by providing additional information not in the ms, or briefly explain why the item is not applicable/relevant for your study

Not applicable.

DISCUSSION

22) Interpretation consistent with results, balancing benefits and harms, and considering other relevant evidence

NPT: In addition, take into account the choice of the comparator, lack of or partial blinding, and unequal expertise of care providers or centers in each group

22-i) Restate study questions and summarize the answers suggested by the data, starting with primary outcomes and process outcomes (use)

Restate study questions and summarize the answers suggested by the data, starting with primary outcomes and process outcomes (use).

	1	2	3	4	5	
subitem not at all important	○	○	○	○	◉	essential

Clear selection

Does your paper address subitem 22-i? *

Copy and paste relevant sections from the manuscript (include quotes in quotation marks "like this" to indicate direct quotes from your manuscript), or elaborate on this item by providing additional information not in the ms, or briefly explain why the item is not applicable/relevant for your study

“Two main findings are noteworthy. First, 12-month outcomes were significantly better for participants randomized to self-guided than either guided wb-CBT or TAU, whereas 12-month outcomes were not better for those randomized to guided wb-CBT than TAU even though 3-month outcomes were best for those randomized to guided wb-CBT. Second, we found that these significant differences were confined to the 40% of participants with the highest predicted compliance with self-guided wb-CBT over weeks 13-52 based on the covariates assessed before randomization. This specification suggests that the superiority of self-guided wb-CBT at 12 months is due to the higher continued use of the self-guided than guided wb-CBT after the guidance ends.”

22-ii) Highlight unanswered new questions, suggest future research

Highlight unanswered new questions, suggest future research.

	1	2	3	4	5	
subitem not at all important	○	○	○	○	◉	essential

Clear selection

Does your paper address subitem 22-ii?

Copy and paste relevant sections from the manuscript (include quotes in quotation marks "like this" to indicate direct quotes from your manuscript), or elaborate on this item by providing additional information not in the ms, or briefly explain why the item is not applicable/relevant for your study

“We are aware of no studies other than our current study that examined the effect of compliance by using baseline covariates to create a measure of predicted compliance. A great appeal of this approach is that it yields valid estimates of heterogeneity in intervention effects with respect to baseline variables that do not rely on the implausible assumptions needed in conventional per-protocol, as-treated, and IV analyses. Another appeal is that our approach provides a principled basis for specifying a precision treatment rule that can be used to help guide allocation of interventions to future patients in a way that maximizes the scalability of treatment. “

20) Trial limitations, addressing sources of potential bias, imprecision, and, if relevant, multiplicity of analyses

20-i) Typical limitations in ehealth trials

Typical limitations in ehealth trials: Participants in ehealth trials are rarely blinded. Ehealth trials often look at a multiplicity of outcomes, increasing risk for a Type I error. Discuss biases due to non-use of the intervention/usability issues, biases through informed consent procedures, unexpected events.

	1	2	3	4	5	
subitem not at all important	○	○	○	○	◉	essential

Clear selection

Does your paper address subitem 20-i? *

Copy and paste relevant sections from the manuscript (include quotes in quotation marks "like this" to indicate direct quotes from your manuscript), or elaborate on this item by providing additional information not in the ms, or briefly explain why the item is not applicable/relevant for your study

“Our study had four noteworthy limitations. First, the study was carried out during the COVID-19 pandemic. It is unclear whether this influenced results. Second, overall intervention compliance was low. This is consistent with many other web-based intervention studies [14] and may be due not only to characteristics of the users but also to characteristics of the programs [52] and might have been exacerbated by the pandemic. Because of this low compliance and context, our results involving interactions between intervention assignment and predicted compliance might not generalize beyond the specific web-based interventions considered here. Third, even though we had a large set of baseline covariates, these variables were chosen as predictors of treatment response rather than of intervention compliance. Future research designed to study the effects of compliance should include a broader set of baseline covariates that include known predictors of compliance [53-55]. Fourth, TAU was heterogeneous across universities and was mainly conducted via videoconferencing because the trial was conducted during the COVID-19 pandemic lockdown. Taken together, these limitations suggest that caution should be taken in assuming that the results generalize beyond the specific time and setting in which the trial was carried out.”

21) Generalisability (external validity, applicability) of the trial findings

NPT: External validity of the trial findings according to the intervention, comparators, patients, and care providers or centers involved in the trial

21-i) Generalizability to other populations

Generalizability to other populations: In particular, discuss generalizability to a general Internet population, outside of a RCT setting, and general patient population, including applicability of the study results for other organizations

	1	2	3	4	5	
subitem not at all important	○	○	○	○	◉	essential

Clear selection

Does your paper address subitem 21-i?

Copy and paste relevant sections from the manuscript (include quotes in quotation marks "like this" to indicate direct quotes from your manuscript), or elaborate on this item by providing additional information not in the ms, or briefly explain why the item is not applicable/relevant for your study

“And we need to consider what interventions to offer students with lower predicted compliance. It would be useful to focus trials of distinct interventions on that segment of the population.”

21-ii) Discuss if there were elements in the RCT that would be different in a routine application setting

Discuss if there were elements in the RCT that would be different in a routine application setting (e.g., prompts/reminders, more human involvement, training sessions or other co-interventions) and what impact the omission of these elements could have on use, adoption, or outcomes if the intervention is applied outside of a RCT setting.

	1	2	3	4	5	
subitem not at all important	○	○	◉	○	○	essential

Clear selection

Does your paper address subitem 21-ii?

Copy and paste relevant sections from the manuscript (include quotes in quotation marks "like this" to indicate direct quotes from your manuscript), or elaborate on this item by providing additional information not in the ms, or briefly explain why the item is not applicable/relevant for your study

“Second, the importance of sustained use of the self-guided wb-CBT platform suggests that learning CBT skills is not enough in itself, but that ongoing practice and review of materials is needed, presumably to help consolidate acquired CBT skills in various life contexts. Participants randomized to guided wb-CBT are trained in the intervention to be extrinsically, instead of intrinsically, motivated to engage with the intervention by virtue of the external reinforcement they receive from their guide, leading to a greater reduction in use of the intervention once guidance ends. This is true, importantly, even for the subset of these participants whose baseline profiles suggest that they would have complied intrinsically if they had been randomized to self-guided wb-CBT. The individuals with this baseline profile who are randomized to self-guided wb-CBT, in comparison, have significantly better longer-term outcomes than if they were randomized to guided wb-CBT, because self-guidance allowed these individuals to consolidate their intrinsic motivation to support longer-term use of the intervention. One implication of this finding is that guided wb-CBT programs need to consider how best to foster intrinsic motivation, possibly through strategies such as intermittent guidance, the use of longer-term booster sessions, tapering guidance over a longer time, guidance-on-demand, or just-in-time adaptive guidance. Although some limited research on such possibilities exists [50, 51], our results suggest that this area warrants further study.”

OTHER INFORMATION

23) Registration number and name of trial registry

Does your paper address CONSORT subitem 23? *

Copy and paste relevant sections from the manuscript (include quotes in quotation marks "like this" to indicate direct quotes from your manuscript), or elaborate on this item by providing additional information not in the ms, or briefly explain why the item is not applicable/relevant for your study

“ClinicalTrials.gov (NCT04780542)”

24) Where the full trial protocol can be accessed, if available

Does your paper address CONSORT subitem 24? *

Cite a Multimedia Appendix, other reference, or copy and paste relevant sections from the manuscript (include quotes in quotation marks "like this" to indicate direct quotes from your manuscript), or elaborate on this item by providing additional information not in the ms, or briefly explain why the item is not applicable/relevant for your study

https://pubmed.ncbi.nlm.nih.gov/35658942/

25) Sources of funding and other support (such as supply of drugs), role of funders

Does your paper address CONSORT subitem 25? *

Copy and paste relevant sections from the manuscript (include quotes in quotation marks "like this" to indicate direct quotes from your manuscript), or elaborate on this item by providing additional information not in the ms, or briefly explain why the item is not applicable/relevant for your study

"This trial is funded by grant number R01MH120648 from the National Institute of Mental Health and Fogarty International Center. The funder had no role in the design, data collection, data analysis, and reporting of this study."

X27) Conflicts of Interest (not a CONSORT item)

X27-i) State the relation of the study team towards the system being evaluated

In addition to the usual declaration of interests (financial or otherwise), also state the relation of the study team towards the system being evaluated, i.e., state if the authors/ /evaluators are distinct from or identical with the developers/sponsors of the intervention.

	1	2	3	4	5	
subitem not at all important	○	○	○	○	◉	essential

Clear selection

Does your paper address subitem X27-i?

Copy and paste relevant sections from the manuscript (include quotes in quotation marks "like this" to indicate direct quotes from your manuscript), or elaborate on this item by providing additional information not in the ms, or briefly explain why the item is not applicable/relevant for your study

The authors are distinct from the developers/sponsors of the intervention. “In the past 3 years, RCK was a consultant for Cambridge Health Alliance, Canandaigua VA Medical Center, Child Mind Institute, Holmusk, Massachusetts General Hospital, Partners Healthcare, Inc., RallyPoint Networks, Inc., Sage Therapeutics and University of North Carolina. He has stock options in Cerebral Inc., Mirah, PYM (Prepare Your Mind), Roga Sciences and Verisense Health. The other authors have no conflicts of interest to declare.”

About the CONSORT EHEALTH checklist

As a result of using this checklist, did you make changes in your manuscript? *

- ○ yes, major changes
- ◉ yes, minor changes
- ○ no

What were the most important changes you made as a result of using this checklist?

More detail in the abstract.

How much time did you spend on going through the checklist INCLUDING making changes in your manuscript *

4 hours.

As a result of using this checklist, do you think your manuscript has improved? *

- (x) yes
- ( ) no
- ( ) Other:

Would you like to become involved in the CONSORT EHEALTH group?

This would involve for example becoming involved in participating in a workshop and writing an "Explanation and Elaboration" document

- ( ) yes
- (x) no
- ( ) Other:

Clear selection

Any other comments or questions on CONSORT EHEALTH

Your answer

STOP - Save this form as PDF before you click submit
To generate a record that you filled in this form, we recommend to generate a PDF of this page (on a Mac, simply select "print" and then select "print as PDF") before you submit it.

When you submit your (revised) paper to JMIR, please upload the PDF as supplementary file.

Don't worry if some text in the textboxes is cut off, as we still have the complete information in our database. Thank you!

Final step: Click submit !
Click submit so we have your answers in our database!

Submit Clear form

Never submit passwords through Google Forms.

This content is neither created nor endorsed by Google. - Terms of Service - Privacy Policy

Does this form look suspicious? Report

Google Forms
