# Supplementary material for: The Effect of Predicted Compliance With a Web-Based Intervention for Anxiety and Depression Among Latin American University Students: Randomized Controlled Trial
Source: JMIR Ment Health. 2025 Feb 28;12:e64251. doi: 10.2196/64251 (PMC11909483; doi:10.2196/64251)
Supplement: Multimedia Appendix 2 [file mental_v12i1e64251_app2.docx]

| **Table S1. Selected baseline characteristics of participants** | | | | | | | | | | | |
| --- | --- | --- | --- | --- | --- | --- | --- | --- | --- | --- | --- |
|  | **Total** | |  | **Guided** | |  | **Self-guided** | |  | **TAU** | |
| Baseline characteristics | ***n*** | **(%)** |  | ***n*** | **(%)** |  | ***n*** | **(%)** |  | ***n*** | **(%)** |
| **I. Country** |  |  |  |  |  |  |  |  |  |  |  |
| Colombia | 594 | (45.0) |  | 203 | (45.6) |  | 191 | (43.5) |  | 200 | (46.0) |
| Mexico | 725 | (55.0) |  | 242 | (54.4) |  | 248 | (56.5) |  | 235 | (54.0) |
| **II. Sex** |  |  |  |  |  |  |  |  |  |  |  |
| Male | 281 | (21.3) |  | 93 | (20.9) |  | 98 | (22.3) |  | 90 | (20.7) |
| Female | 1,038 | (78.7) |  | 352 | (79.1) |  | 341 | (77.7) |  | 345 | (79.3) |
| **III. Sexual orientation** |  |  |  |  |  |  |  |  |  |  |  |
| Heterosexual | 916 | (69.4) |  | 308 | (69.2) |  | 293 | (66.7) |  | 315 | (72.4) |
| Gay or lesbian | 61 | (4.6) |  | 26 | (5.8) |  | 17 | (3.9) |  | 18 | (4.1) |
| Bisexual | 196 | (14.9) |  | 55 | (12.4) |  | 80 | (18.2) |  | 61 | (14.0) |
| Other (asexual, unsure, "other") | 146 | (11.1) |  | 56 | (12.6) |  | 49 | (11.2) |  | 41 | (9.4) |
| **IV. Age** |  |  |  |  |  |  |  |  |  |  |  |
| 18-19 | 354 | (26.8) |  | 124 | (27.9) |  | 98 | (22.3) |  | 132 | (30.3) |
| 20 | 251 | (19.0) |  | 76 | (17.1) |  | 105 | (23.9) |  | 70 | (16.1) |
| 21-22 | 397 | (30.1) |  | 130 | (29.2) |  | 138 | (31.4) |  | 129 | (29.6) |
| 23 or older | 317 | (24.0) |  | 115 | (25.8) |  | 98 | (22.3) |  | 104 | (23.9) |
| **V. First-generation university student** |  |  |  |  |  |  |  |  |  |  |  |
| Yes | 738 | (55.9) |  | 252 | (56.6) |  | 240 | (54.7) |  | 246 | (56.5) |
| No | 581 | (44.0) |  | 193 | (43.4) |  | 199 | (45.3) |  | 189 | (43.4) |
| **VI. Did the university have a mental health clinic?** |  |  |  |  |  |  |  |  |  |  |  |
| Yes and student recruited from waiting list | 290 | (22.0) |  | 97 | (21.8) |  | 98 | (22.3) |  | 95 | (21.8) |
| Yes but student recruited from student body | 607 | (46.0) |  | 207 | (46.5) |  | 200 | (45.6) |  | 200 | (46.0) |
| No (all students recruited from student body) | 422 | (32.0) |  | 141 | (31.7) |  | 141 | (32.1) |  | 140 | (32.2) |
| **VII. Severity of anxiety (GAD-7)^a^** |  |  |  |  |  |  |  |  |  |  |  |
| Severe | 494 | (37.4) |  | 164 | (36.8) |  | 164 | (37.4) |  | 166 | (38.2) |
| Moderate | 434 | (32.9) |  | 149 | (33.5) |  | 146 | (33.3) |  | 139 | (31.9) |
| Mild or none | 391 | (29.6) |  | 132 | (29.7) |  | 129 | (29.4) |  | 130 | (29.9) |
| **VIII. Severity of depression (PHQ-9)^b^** |  |  |  |  |  |  |  |  |  |  |  |
| Severe | 488 | (37.0) |  | 160 | (35.9) |  | 159 | (36.2) |  | 169 | (38.8) |
| Moderate (including moderate-severe) | 702 | (53.2) |  | 240 | (53.9) |  | 236 | (53.8) |  | 226 | (51.9) |
| Mild or none | 129 | (9.8) |  | 45 | (10.1) |  | 44 | (10.0) |  | 40 | (9.2) |
| **IX. Comorbidity** |  |  |  |  |  |  |  |  |  |  |  |
| Severe^c^ on both GAD-7 and PHQ-9 | 282 | (21.4) |  | 95 | (21.3) |  | 95 | (21.6) |  | 92 | (21.1) |
| Severe^c^ on one and moderate on the other | 284 | (21.5) |  | 88 | (19.8) |  | 88 | (20.0) |  | 108 | (24.8) |
| Severe^c^ on one and mild–none on the other | 134 | (10.2) |  | 46 | (10.3) |  | 45 | (10.2) |  | 43 | (9.9) |
| Moderate on both | 233 | (17.7) |  | 85 | (19.1) |  | 83 | (18.9) |  | 65 | (14.9) |
| Moderate on one and mild–none on the other | 386 | (29.3) |  | 131 | (29.4) |  | 128 | (29.2) |  | 127 | (29.2) |
|  |  |  |  |  |  |  |  |  |  |  |  |
| (*n*) | (1,319) | |  | (445) | |  | (439) | |  | (435) | |
|  |  |  |  |  |  |  |  |  |  |  |  |

Abbreviations: Guided, guided web-based cognitive behavioral therapy (wb-CBT); self-guided, self-guided wb-CBT; TAU, treatment as usual; *n*, number of participants defined by the row headings who were in the total randomized baseline sample or in intervention arms; %, the percent of all randomized baseline respondents in the column who were in the subgroups defined by the row headings; GAD-7, 0–21 Generalized Anxiety Disorder–7; PHQ-9, 0–27 Patient Health Questionnaire–9.

^a^Severe refers to GAD-7, 15+, moderate to GAD-7, 10–14, mild or none to GAD-7, 0–9.

^b^Severe refers to PHQ-9, 20+, moderately severe to PHQ-9, 15–19, moderate to PHQ-9, 10–14, mild or none to PHQ-9, 0–9.

^c^Including either severe or moderately severe PHQ-9.

| **Table S2. Interaction between sexual orientation and intervention arms to predict 3-month SAQ completion status (regardless of 12-month SAQ completion status) (*n* = 1,319)** | | | | | | | | |
| --- | --- | --- | --- | --- | --- | --- | --- | --- |
|  | **Guided** | |  | **Self-guided** | |  | **TAU** | |
|  | ***n*** | **(%)** |  | ***n*** | **(%)** |  | ***n*** | **(%)** |
| **Sexual orientation** |  |  |  |  |  |  |  |  |
| Heterosexual | 193 | (62.7) |  | 180 | (61.4) |  | 237 | (75.2) |
| Gay or lesbian | 15 | (57.7) |  | 11 | (64.7) |  | 13 | (72.2) |
| Bisexual | 43 | (78.2) |  | 43 | (53.8) |  | 55 | (90.2) |
| Other (asexual, unsure, "other") | 40 | (71.4) |  | 39 | (79.6) |  | 30 | (73.2) |
| (*n*) | (445) | |  | (439) | |  | (435) | |
|  |  |  |  |  |  |  |  |  |

Abbreviations: SAQ, self-administered questionnaire; *n*, number of baseline participants who completed the follow-up SAQ; %, the proportion of baseline respondents represented by *n*; Guided, guided web-based cognitive behavioral therapy (wb-CBT); Self-guided, self-guided wb-CBT; TAU, treatment as usual.

| **Table S3. Follow-up rates at 3-month and 12-month follow-ups** | | | | | | | | | | | |
| --- | --- | --- | --- | --- | --- | --- | --- | --- | --- | --- | --- |
|  | **Guided** | |  | **Self-guided** | |  | **TAU** | |  | **Difference^a^** | |
| **I. Follow-up rates in the total sample by arm and follow-up period** | | | | | | | | |  |  | |
|  | ***n*** | **(%)** |  | ***n*** | **(%)** |  | ***n*** | **(%)** |  | **χ^2^** | ***P*** |
| 3-month |  |  |  |  |  |  |  |  |  | 1.7 | .42 |
| High predicted compliance subsample | 120 | (68.6) |  | 127 | (65.1) |  | 132 | (83.5) |  |  |  |
| Low predicted compliance subsample | 171 | (63.3) |  | 146 | (59.8) |  | 203 | (73.3) |  |  |  |
| Total | 291 | (65.4) |  | 273 | (62.2) |  | 335 | (77.0) |  |  |  |
| 12-month |  |  |  |  |  |  |  |  |  | 0.8 | .66 |
| High predicted compliance subsample | 122 | (69.7) |  | 127 | (65.1) |  | 120 | (75.9) |  |  |  |
| Low predicted compliance subsample | 169 | (62.6) |  | 153 | (62.7) |  | 191 | (69.0) |  |  |  |
| Total | 291 | (65.4) |  | 280 | (63.8) |  | 311 | (71.5) |  |  |  |
| **II. 12-month follow-up rates among subgroups with or without 3-month completed SAQ responses** | | | | | | | | | | | |
|  | ***n*** | **(%)** |  | ***n*** | **(%)** |  | ***n*** | **(%)** |  | **χ^2^** | ***P*** |
| Completers |  |  |  |  |  |  |  |  |  | 3.0 | .22 |
| High predicted compliance subsample | 103 | (85.8) |  | 103 | (81.1) |  | 102 | (77.3) |  |  |  |
| Low predicted compliance subsample | 131 | (76.6) |  | 117 | (80.1) |  | 160 | (78.8) |  |  |  |
| Total | 234 | (80.4) |  | 220 | (80.6) |  | 262 | (78.2) |  |  |  |
| Non-completers |  |  |  |  |  |  |  |  |  | 5.3 | .07 |
| High predicted compliance subsample | 19 | (34.5) |  | 24 | (35.3) |  | 18 | (69.2) |  |  |  |
| Low predicted compliance subsample | 38 | (38.4) |  | 36 | (36.7) |  | 31 | (41.9) |  |  |  |
| Total | 57 | (37.0) |  | 60 | (36.1) |  | 49 | (49.0) |  |  |  |
| **III. 12-month follow-up rate among 3-month respondents with and without 3-month joint remission** | | | | | | | | | | | |
|  | ***n*** | **(%)** |  | ***n*** | **(%)** |  | ***n*** | **(%)** |  | **χ^2^** | ***P*** |
| Remitters |  |  |  |  |  |  |  |  |  | 2.4 | .30 |
| High predicted compliance subsample | 59 | (85.5) |  | 41 | (82.0) |  | 45 | (80.4) |  |  |  |
| Low predicted compliance subsample | 59 | (76.6) |  | 45 | (84.9) |  | 63 | (85.1) |  |  |  |
| Total | 118 | (80.8) |  | 86 | (83.5) |  | 108 | (83.1) |  |  |  |
| Non-remitters |  |  |  |  |  |  |  |  |  | 1.3 | .52 |
| High predicted compliance subsample | 44 | (86.3) |  | 62 | (80.5) |  | 57 | (75.0) |  |  |  |
| Low predicted compliance subsample | 72 | (76.6) |  | 72 | (77.4) |  | 97 | (75.2) |  |  |  |
| Total | 116 | (80.0) |  | 134 | (78.8) |  | 154 | (75.1) |  |  |  |
|  |  |  |  |  |  |  |  |  |  |  |  |

Abbreviations: Guided, guided web-based cognitive behavioral therapy (wb-CBT); self-guided, self-guided wb-CBT; TAU, treatment as usual; *n*, number of participants defined by the row headings who were in the total randomized baseline sample or in intervention arms; %, the percent of all randomized baseline respondents in the column who were in the subgroups defined by the row headings; *P*, *P*-value; SAQ, self-administered questionnaire.

^a^This is a 2-degree of freedom (*df*) test for the significance of differences across all three arms.

| **Table S4. Average minutes spent on wb-CBT per week by arm** | | | | | | | | |
| --- | --- | --- | --- | --- | --- | --- | --- | --- |
|  | **Guided** | |  | **Self-guided** | |  | **Difference^a^** | |
|  | **Est** | **(SE)** |  | **Est** | **(SE)** |  | **χ^2^** | ***P*** |
| **Weeks 1–12 of assessment** |  |  |  |  |  |  |  |  |
| Average minutes per week | 12.5 | (0.5) |  | 5.9 | (0.4) |  | 107.1* | <.001 |
| % with ≥ 5 minutes per week | 21.1 | (0.6) |  | 9.7 | (0.4) |  | 268.4* | <.001 |
| % with ≥ 10 minutes per week | 18.6 | (0.5) |  | 8.5 | (0.4) |  | 235.6* | <.001 |
| % with ≥ 30 minutes per week | 13.0 | (0.5) |  | 5.7 | (0.3) |  | 171.4* | <.001 |
| **Weeks 13-52 of assessment** |  |  |  |  |  |  |  |  |
| Average minutes per week | 0.2 | (0.0) |  | 0.4 | (0.1) |  | 10.5* | .001 |
| % with ≥ 5 minutes per week | 0.5 | (0.1) |  | 0.8 | (0.1) |  | 17.2* | <.001 |
| % with ≥ 10 minutes per week | 0.4 | (0.0) |  | 0.7 | (0.1) |  | 14.1* | <.001 |
| % with ≥ 30 minutes per week | 0.2 | (0.0) |  | 0.4 | (0.0) |  | 5.3* | .02 |
|  |  |  |  |  |  |  |  |  |

Abbreviations: wb-CBT, web-based cognitive behavioral therapy; Est, estimate of means of minutes spent on guided or self-guided wb-CBT or number of participants within the specific compliance subgroup; SE, standard error; *P*, *P*-value.

^a^This is a 1-degree of freedom (*df*) test for the significance of differences between guided and self-guided wb-CBT arms.

| **Table S5. Average treatment effects across arms at 3 months with the ARDs for joint remission and the AMDs for mean PHQ-ADS scores stratified by predicted compliance with self-guided wb-CBT in weeks 13-52** | | | | | | | | | | | |
| --- | --- | --- | --- | --- | --- | --- | --- | --- | --- | --- | --- |
|  | **Intervention arm** | | | | | | | | | | |
|  | **Total** | |  | **Guided** | |  | **Self-guided** | |  | **TAU** | |
| **I. Joint remission of GAD-7 and PHQ-9^a^** |  | |  |  | |  |  | |  |  | |
| **A. Rates** | **Est** | **(SE)** |  | **Est** | **(SE)** |  | **Est** | **(SE)** |  | **Est** | **(SE)** |
| High predicted compliance subsample^b^ | 46.5 | (8.0) |  | 57.6 | (4.6) |  | 39.3 | (4.4) |  | 42.5 | (4.4) |
| Low predicted compliance subsample^c^ | 30.9 | (7.0) |  | 45.1 | (3.8) |  | 36.1 | (4.0) |  | 36.5 | (3.5) |
|  |  |  |  |  |  |  |  |  |  |  |  |
| **B. *ARD* in the high predicted compliance subsample^b^** | **ARD** | **(SE)** |  | **ARD** | **(SE)** |  | **ARD** | **(SE)** |  | **ARD** | **(SE)** |
| Versus self-guided wb-CBT | - | - |  | 18.3* | (6.3) |  | - | - |  | 3.2 | (6.2) |
| Versus TAU | - | - |  | 15.1* | (6.3) |  | -3.3 | (6.2) |  | - | - |
|  |  |  |  |  |  |  |  |  |  |  |  |
| **C. *ARD* in the lower predicted compliance subsample^c^** | **ARD** | **(SE)** |  | **ARD** | **(SE)** |  | **ARD** | **(SE)** |  | **ARD** | **(SE)** |
| Versus self-guided wb-CBT | - | - |  | 9.0 | (5.5) |  | - | - |  | 0.4 | (5.3) |
| Versus TAU | - | - |  | 8.6 | (5.1) |  | -0.4 | (5.3) |  | - | - |
|  |  |  |  |  |  |  |  |  |  |  |  |
| **II. Mean PHQ-ADS scores** |  |  |  |  |  |  |  |  |  |  |  |
| **A. Means** | **Est** | **(SE)** |  | **Est** | **(SE)** |  | **Est** | **(SE)** |  | **Est** | **(SE)** |
| High predicted compliance subsample^b^ |  |  |  |  |  |  |  |  |  |  |  |
| Baseline | 26.9 | (2.7) |  | 26.7 | (1.5) |  | 26.7 | (1.5) |  | 27.3 | (1.6) |
| 3-month | 13.6 | (1.9) |  | 11.8 | (1.1) |  | 14.0 | (1.0) |  | 14.9 | (1.1) |
| Low predicted compliance subsample^c^ |  |  |  |  |  |  |  |  |  |  |  |
| Baseline | 29.8 | (2.3) |  | 30.2 | (1.4) |  | 29.4 | (1.4) |  | 29.7 | (1.2) |
| 3-month | 15.3 | (1.6) |  | 13.9 | (0.9) |  | 16.0 | (1.0) |  | 16.1 | (0.9) |
|  |  |  |  |  |  |  |  |  |  |  |  |
| **B. *AMD* in the high predicted compliance subsample^b^** | **AMD** | **(SE)** |  | **AMD** | **(SE)** |  | **AMD** | **(SE)** |  | **AMD** | **(SE)** |
| Versus self-guided wb-CBT | - | - |  | -2.2 | (1.5) |  | - | - |  | 1.0 | (1.5) |
| Versus TAU | - | - |  | -3.1 | (1.6) |  | -1.0 | (1.5) |  | - | - |
|  |  |  |  |  |  |  |  |  |  |  |  |
| **C. *AMD* in the lower predicted compliance subsample^c^** | **AMD** | **(SE)** |  | **AMD** | **(SE)** |  | **AMD** | **(SE)** |  | **AMD** | **(SE)** |
| Versus self-guided wb-CBT | - | - |  | -2.0 | (1.4) |  | - | - |  | 0.1 | (1.3) |
| Versus TAU | - | - |  | -2.1 | (1.3) |  | -0.1 | (1.3) |  | - | - |
| (*n*_1_)^b^ | (528) | |  | (175) | |  | (195) | |  | (158) | |
| (*n*_2_)^c^ | (791) | |  | (270) | |  | (244) | |  | (277) | |
|  |  |  |  |  |  |  |  |  |  |  |  |

Abbreviations: ARD, adjusted risk difference; AMD, adjusted mean difference; PHQ-ADS, 0–48 Patient Health Questionnaire Anxiety and Depression Scale; Guided, guided web-based cognitive behavioral therapy (wb-CBT); Self-guided, self-guided wb-CBT; TAU, treatment as usual; GAD-7, 0–21 Generalized Anxiety Disorder–7; PHQ-9, 0–27, Patient Health Questionnaire–9; *n*, number of participants defined by the row headings who were in the total randomized baseline sample or in intervention arms; n^1^, predicted high compliance; n^2^, predicted lower compliance; Est, estimate of joint remission rates of both GAD-7 and PHQ-9 or means of PHQ-ADS scores; SE, standard error of AMD, ARD, or Est; ATE, average treatment effect; n_1_, number of participants with high predicted compliance; n_2_, number of participants with lower predicted compliance.

^a^Joint remission was defined as scores of 0–4 on both the GAD-7 and the PHQ-9.

^b^For the high predicted compliance subsample, the 2-degree of freedom (*df*) at the .05 level, two-sided test for the overall variations across the three arms on joint remission of GAD-7 and PHQ-9 were statistically significant at 3 months (χ^2^_2_ = 9.5, *P*=.009). In addition, the overall variations across the three arms on mean PHQ-ADS scores were not statistically significant at baseline (χ^2^_2_ = 0.3, *P*=.87) and 3 months (χ^2^_2_ = 4.2, *P*=.12) among participants with low predicted compliance.

^c^For the low predicted compliance subsample, the 2-*df* at the .05 level, two-sided test for the overall variations across the three arms on joint remission of GAD-7 and PHQ-9 were not statistically significant at 3 months (χ^2^_2_ = 3.6, *P*=.16). In addition, the overall variations across the three arms on mean PHQ-ADS scores were not statistically significant at baseline (χ^2^_2_ = 0.6, *P*=.73) and 3 months (χ^2^ = 3.4, *P*=.18) among participants with high predicted compliance.

* Significant difference in ARD or AMD between the arm in the row heading and the arm in the column heading at the .05 level, two-sided test.
